# Genome-wide identification of actin-depolymerizing factor family genes in melon (*Cucumis melo* L.) and CmADF1 plays an important role in low temperature tolerance

**DOI:** 10.3389/fpls.2024.1419719

**Published:** 2024-08-22

**Authors:** Yanling Lv, Shihang Liu, Jiawang Zhang, Jianing Cheng, Jinshu Wang, Lina Wang, Mingyang Li, Lu Wang, Shuangtian Bi, Wei Liu, Lili Zhang, Shilei Liu, Dabo Yan, Chengxuan Diao, Shaobin Zhang, Ming He, Yue Gao, Che Wang

**Affiliations:** ^1^ College of Bioscience and Biotechnology, Shenyang Agricultural University, Shenyang, China; ^2^ College of Horticulture, Shenyang Agricultural University, Shenyang, China; ^3^ Institute of Vegetable, Liaoning Academy of Agricultural Sciences, Shenyang, China

**Keywords:** genome-wide identification, CmADF1, low temperature, oriental melon, Arabidopsis

## Abstract

Actin depolymerizing factors (ADFs), as the important actin-binding proteins (ABPs) with depolymerizing/severing actin filaments, play a critical role in plant growth and development, and in response to biotic and abiotic stresses. However, the information and function of the ADF family in melon remains unclear. In this study, 9 melon ADF genes (CmADFs) were identified, distributed in 4 subfamilies, and located on 6 chromosomes respectively. Promoter analysis revealed that the CmADFs contained a large number of cis-acting elements related to hormones and stresses. The similarity of CmADFs with their Arabidopsis homologue AtADFs in sequence, structure, important sites and tissue expression confirmed that ADFs were conserved. Gene expression analysis showed that CmADFs responded to low and high temperature stresses, as well as ABA and SA signals. In particular, CmADF1 was significantly up-regulated under above all stress and hormone treatments, indicating that CmADF1 plays a key role in stress and hormone signaling responses, so CmADF1 was selected to further study the mechanism in plant tolerance low temperature. Under low temperature, virus-induced gene silencing (VIGS) of CmADF1 in oriental melon plants showed increased sensitivity to low temperature stress. Consistently, the stable genetic overexpression of CmADF1 in Arabidopsis improved their low temperature tolerance, possibly due to the role of CmADF1 in the depolymerization of actin filaments. Overall, our findings indicated that CmADF genes, especially CmADF1, function in response to abiotic stresses in melon.

## Introduction

Melon (*Cucumis melo* L.), originated from tropical zone, is a worldwide fruit with high edible value and economic value, and is sensitive to low temperature. Under the influence of the environment and climate in the northern region, the oriental melon cultivated in facilities in winter and spring is often affected by low temperature, which seriously deteriorates their edible quality and commercial value, and even no harvest (Zhang et al., 2017). Low temperature has become an important limiting factor for facility cultivation of oriental melon in winter and spring.

In the process of resisting unfavorable conditions, plants have evolved strategies to protect themselves (Ding et al., 2019). The cytoskeleton is closely related to various environmental stimuli (Wang et al., 2011; Sengupta et al., 2019; Byun et al., 2021). Actin filaments are a major member of cytoskeleton and play an important role in stress responses (Zhang et al., 2010; Wang et al., 2011; Ye et al., 2013; Fan et al., 2016). Depolymerization of actin filaments in *Arabidopsis* plants under salt and osmotic stress can improve plant tolerance (Zhang et al., 2010; Wang et al., 2011; Ye et al., 2013). Pokorná et al. (2004) found that in 3-day-old BY-2 cells exposed at 0°C for 12 hours, actin filaments disintegrated completely, or turned into few in number, short, and sometimes branched filaments, actin bars or dots. The findings of Fan et al. (2015; 2016) suggest that actin cytoskeleton plays a key role in the tolerance of *Arabidopsis* seedlings to low temperature and heat stress, and specific members of actin depolymerizing factors (ADFs) may be involved in regulating plant response to low temperature and heat stress. Destabilizers of actin filaments and microtubules cause the activation of cold-inducible *Brassica napus BN115* (Sangwan et al., 2001). However, the molecular mechanism of the dynamic change of actin filaments under low temperature is poorly understood.

The dynamic reorganization of intracellular actin filaments is regulated by a large number of ABPs with different functions (Hussey et al., 2006; Huang et al., 2011; Roland et al., 2008), in which ADFs are considered to be an important regulator of actin filaments changes (Maciver and Hussey, 2002; Bamburg and Bernstein, 2008). ADF is abundant and highly conserved in all eukaryotes, and plays an important role in plant growth and development as well as in response to multiple biotic and abiotic stresses (Andrianantoandro and Pollard, 2006; Dong et al., 2001, 2013). The functions of ADFs in Arabidopsis have been studied *in vivo* extensively. For example, AtADF1 can affect plants growth, development and morphogenesis (Dong et al., 2001), and participate in high temperature and salt stress processes (Wang et al., 2021, 2023). AtADF2 is required for normal cell growth and plant development, and its mediated actin dynamic is essential for root-knot nematode infection of Arabidopsis (Clément et al., 2009). AtADF4 relates to plants growth and development (Peng and Huang, 2006), plays a role in regulating hypocotyl growth, response to osmotic (Yao et al., 2022) and drought stresses (Zhao et al., 2016), and improves disease resistance of *Arabidopsis* to bacterium DC3000AvrPphB (Tian et al., 2009). AtADF5 is important for pollen germination and pollen tube growth (Zhu et al., 2017), promotes stomatal closure by regulating actin cytoskeleton remodeling under ABA and drought stresses (Qian et al., 2019), and improves the basal and acquired freezing resistance of *Arabidopsis* (Zhang et al., 2021). AtADF7 and AtADF10 are involved in pollen development and pollen tube growth (Daher and Geitmann, 2012; Zheng et al., 2013). Under osmotic stress, AtADF7 inhibited actin bundling protein VILLIN1 regulation of root hair formation (Bi et al., 2022). In addition, ADFs from barley and wheat have been shown to be related to plant resistance to various pathogens (Miklis et al., 2007; Fu et al., 2014; Inada et al., 2016). TaADF4 and TaADF7 from wheat play a stimulative role in resistance to the stripe rust infection (Zhang et al., 2017; Fu et al., 2014). TaADF3 negatively regulates wheat resistance against Puccinia striiformis (Tang et al., 2016). Increasing evidence has shown that ADFs play an important role in response and tolerance to various stresses (Huang et al., 2012; Xu et al., 2021). Drought resistance of *OsADF3* in rice transgenic *Arabidopsis* is enhanced (Huang et al., 2012). AtADF5 improves the basal and acquired freezing resistance of *Arabidopsis* (Zhang et al., 2021). Overexpression of *TaADF16* significantly improved the tolerance of transgenic plants to freezing stress (Xu et al., 2021). DaADF3 in *Deschampsia antarctica* enhanced the cold tolerance of transgenic rice plants (Byun et al., 2021). In the process of wheat cold acclimation, an *ADF* gene is induced, and the increased resistance to freezing shows that the ADF protein may be required in reorganization of the cytoskeleton under low temperatures (Ouellet et al., 2001). In short, more and more plant ADFs have been functionally characterized, while ADFs in oriental melon have not been reported.

In this study, we identified 9 *CmADF* genes in oriental melon and found that they were similar to homologue *AtADF* genes in sequence, structure, important site and tissue expression through analysis of their biological information and tissue expression. Further stress expression patterns showed that CmADFs responded to low temperature, high temperature, ABA and SA signals, especially under SA treatment, all CmADFs were dramatically up-regulated by approximately ten to hundreds of times. *CmADF1* was significantly upregulated under all the above treatments, especially during 24 h of low temperature treatment, and maintained high expression, which provides the functional implication of CmADF1 in low temperature response. Further studies on the phenotype and actin filaments organization of *Arabidopsis* seedlings overexpressing *CmADF1* under low temperature, as well as phenotype analysis and physiological identification of *CmADF1* gene silenced oriental melon seedlings, indicated that CmADF1 affected the process of actin filaments depolymerization and played an important role in plant adaptation to low temperature stress.

## Materials and methods

### Plant materials, growth conditions, and stress treatments

The low temperature tolerant genotype Oriental melon ‘ LT-6 ‘ was provided by the Vegetable Research Institute of Liaoning Academy of Agricultural Sciences, and the *CmADF1* silent plant was obtained by VIGS technology. The Oriental melon grew at 25/20°C (light 16 h/darkness 8 h) to two-leaf stage and was subjected to low temperature (4°C), high temperature (40°C), ABA (100 μM) and SA (100 μM) treatment for stress expression analysis. The second true leaves were sampled at 0, 3, 6, 12 and 24 h after treatment. Tissues of two-month-old plants, including roots, stems, young leaves, pistillate and staminate flowers, were collected for tissue expression analysis. The silenced plants of *CmADF1* (TRV-A) were treated at 4°C at the two-leaf stage to observe the phenotype and analyze the physiological indexes.

The *Arabidopsis thaliana* plants used in this study have a Columbia background. *Atadf1* (The T-DNA insertion mutant) (SALK_144459) was obtained from ABRC, and the *fABD2-GFP* material was donated by China Agricultural University (Wang et al., 2021). The *Arabidopsis* overexpression materials (*CmADF1*-OE and *pCmADF1*::GUS) were constructed by our laboratory. The seeds of WT and *CmADF1*-OE with 4°C vernalization for 3 days were seeded on 1/2 MS medium (Wang et al., 2023), and cultured in a 22°C incubator with a light/dark cycle of 16 h/8 h. After 14 days, the seedlings were placed in incubators at 22°C and 4°C for 0, 24 and 48h respectively, and the leaf area was counted by Image J software. WT and *CmADF1*-OE plants grown under normal conditions for 9 days were treated at 4°C for 12 h, and the morphology of actin filaments in leaves was observed.

### Identification of the *ADF* gene family in oriental melon

To identify the *ADF* gene family members in oriental melon, amino acid sequences of 11 *ADFs* in *Arabidopsis* with ADF-H (Actin-Depolymerizing Factor Homology) domain were used as query sequences to search against the entire melon genome database (http://melonomics.net/) with the threshold E≤e^-20^ and default parameters by performing a BLASTP analysis. Then, the candidate sequences of ADF proteins in the melon genome were used repeatedly to search new ADFs. The longest protein sequence were selected when there were more than one predicted ADF proteins resulting from the alternative splicing by one gene. All identified ADF proteins were checked if they contained ADF-H domain by SMART (http://smart.embl-heidelberg.de/) analysis and hidden Markoy model analysis with PF00241 (http://pfam.xfam.org/family/PF00241). The protein sequences and genome sequences of CmADFs are downloaded from the Melon Genome database. CmADF paralogous genes to AtADF were named for the corresponding AtADFs. Molecular weight (kDa) and isoelectric points (pI) were calculated by the pI/Mw tool at online ProtParam (http://web.expasy.org/protparam/). Subcellular localization was predicted by WoLF PSORT (https://www.genscript.com/tools/psort). We predicted the tertiary structure of the CmADF protein using SWISS-MODEI (http://swissmodel.expasy.org/) and displayed the images using PyMOL V2.3.2 software. The genomic DNA sequence of 2000 bp upstream of gene initiation codon (ATG) was used as the promoter sequence and submitted to the promoter analysis system PlantCARE (http://bioinformatics.psb.ugent.Be/webtools/plantcare/html/) to find all potential cis-acting elements.

### Sequence alignment and phylogenetic analysis

The ADF amino acid sequences of melon, *Arabidopsis*, cucumber, watermelon, *Cucurbita maxima* and *Cucurbita pepo* were aligned using ClustalX 2.1 (Larkin et al., 2007). ESpript3.0 (http://espript.ibcp.fr/ESPript/cgi-bin/ESPript.cgi) was used for image display. The unrooted phylogenetic tree was generated in MEGA6.0 using the neighbor-joining (NJ) method with 1000 bootstrap replicates (Tamura et al., 2013).

### Chromosome localization and gene duplication

The chromosomal locations for each *CmADF* were determined according to melon genome information. Tandem and segmental duplications were determined using CoGe (https://genomevolution.org/CoGe/) online tool. Duplicated genes were linked using Circos software (http://circos.ca/). The non-synonymous substitution rate (Ka) and synonymous substitution rate (Ks) were calculated by DnaSp V5.0 (Librado and Rozas, 2009) software. The approximate time of each duplication event (T=Ks/2λ, λ=6.1×10^-9^) (Lynch and Conery, 2000) was estimated by Ks value.

### Gene structure and conserved motif analysis of ADF in melon and *Arabidopsis*

According to structural information of *ADF* genes obtained from the gff3 files of melon and *Arabidopsis* genome databases, the intron-exon structures of each gene were drawn using GSDS (http://gsds.cbi.pku.edu.cn) online tool. MEME4.10.2 software (http://meme-suite.Org/tools/meme) was used to analyze the conserved motifs of *ADF* genes in melon and *Arabidopsis*. The maximum value of searched motif was set to 10, the length of motif was between 6 and 50, and other parameters were default values.

### Total RNA extraction and real-time fluorescence quantitative PCR analysis

All total RNA was extracted by EasyPure Plant RNA Kit (Beijing Quanshi Jin Biotechnology Co., LTD.). 18S was selected as the internal control for RT-qPCR analysis. The primer sequences are shown in **Supplementary Table 3**. Roche Light Cycler 480 was used to detect the relative expression level of *ADFs*.

### Overexpression and subcellular localization of CmADF1

The full-length CDS of *CmADF1* was respectively cloned into the pSuper1300-GFP and pCAMBIA 1300 vectors to generate *35S::CmADF1*-GFP and *35S::CmADF1*, which were then introduced into the Agrobacterium tumefaciens strain GV3101. The primer sequences are listed in **Supplementary Table 3**. T3 transgenic homozygous lines were screened for confocal microscope observation and low temperature study. *35S::CmADF1*-GFP was used to observe subcellular localization. *35S::CmADF1*was used in subsequent low temperature stress studies.

### Promoter activity analysis

*CmADF1* promoter fragment containing the first intron of *CmADF1* was cloned in pCAMBIA1300-221 vector to generate *pADF1*::GUS. Homozygous lines were used for promoter activity analysis. The positive seedlings growing for 9 days were treated at 4 °C for 0, 6, and 12 h, and GUS staining was performed. The primer sequences are listed in **Supplementary Table 3**.

### Visualization and quantitative analysis of actin filaments

As previously reported, *CmADF1*-OE#8 plants were hybridized with *fABD2-GFP* plants, and homozygous plants were selected for subsequent experiments. Confocal microscopy (Nikon A1) with a 40×objective was used to observe actin filaments in pavement cells of cotyledons with a 488-nm laser. Image J was used to measure skewness, density, length and actin cables applied to quantify actin filaments in previous reports. All experiments were repeated 3 times. 30 individual seedlings from different genotypes and treatments were screened to collect more than 200 cells from 60 images.

### Western blot assays

10-day-old *CmADF1-GFP* overexpressing Arabidopsis seedlings were used for Western Blot. As previously reported (Liu et al., 2013; Wang et al., 2020), GFP (a labeled protein) was analyzed by SDS-PAGE. Rubisco bands were used as loading controls.

### Vector construction and infection of virus-induced gene silencing

The CmADF1 gene in oriental melon were silenced by VIGS. The specific primers containing EcoRI/KpnI cleavage sites were designed to generate *pTRV2-CmADF1* (**Supplementary Table 3** for the sequence of primers). The method of cotyledon infection was used. The cotyledons of the germinated oriental melon seeds were fully expanded about 5 days after sowing, and they could be infected. The detailed infection process was carried out according to the method of Liao et al. (2019). About 60 infected plants were randomly divided into 3 groups, and each plant was sampled separately after treatment at 4°C for 0, 6 and 12 h. The expression of *CmADF1* in the leaves of VIGS plants was detected by agarose gel electrophoresis and RT-qPCR, and the plants with transcription level lower than 50% of that of the control plants were selected for subsequent experiments.

### Determination of physiological and biochemical indexes and water loss rate

Relative electrolyte leakage (REL) and water loss was measured as described previously (Xing et al., 2020). The contents of soluble protein, proline, malondiald ehyde (MDA) and the activities of SOD, CAT and POD were determined with the relevant kit of Suzhou Mengxi Biomedical Technology Co., LTD. All experiments were repeated three times.

## Results

### Genome-wide identification and bioinformatics analysis of ADF gene family members in oriental melon

The ADF family member from *Arabidopsis* with the ADF-H domain (PF00241) was used for Blast search in the entire melon genome. 11 non-redundant putative ADF proteins were found, 3 of them were produced by one gene, which was subject to differential splicing, so we selected the longest protein sequence. Finally, 9 *CmADF* genes were identified by SMART and Pfam analysis, and they were named with the same names as their homologue *AtADF* genes. CmADF proteins contain 132 (CmADF8) to 146 (CmADF6) amino acids, and their physicochemical properties are shown in **Supplementary Table 1**.

**Figure 1:**
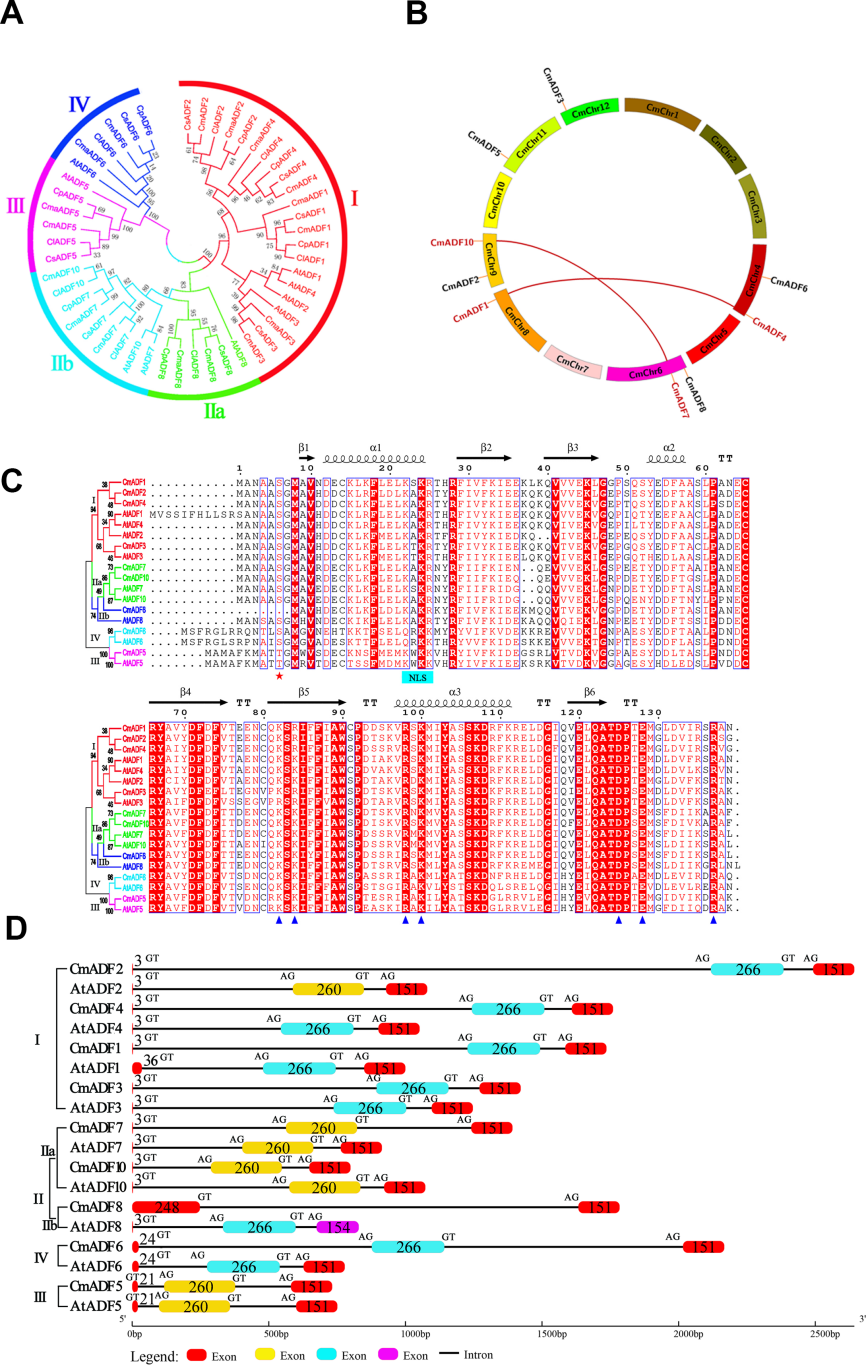
Phylogenetic relationship, Chromosomal location and duplication events, Sequence alignment, exon-intron structures of *CmADFs*. **(A)** Phylogenetic tree of ADF proteins from melon and other plants. Cucumis melon, *Arabidopsis thaliana*, *Cucumis sativus*, *Citrullus lanatus subsp*, *Cucurbita maxima var*, *Cucurbita pepo subsp* were represented by Cm, At, Cs, Cla, Cma and Cp, respectively. **(B)** Location and duplication events of 9 *CmADF* genes onto six melon chromosomes. The segmentally duplicated genes are linked by red lines. **(C)** Sequence alignment of CmADF and AtADF proteins. Pentagons indicate conserved sites that may bind to G-actin and F-actin, blue pentagons indicate the 6th serine site, and blue bar indicates the nuclear localization signal. **(D)** Exon-intron structures of *ADFs* in melon and *Arabidopsis*. The numbers indicates the length of exon. GT and AG indicate splicing sites.

To clarify the phylogenetic relationships and functional divergence of *CmADF* gene family members, amino acid sequences of ADF from *Arabidopsis*, melon and other Cucurbitaceae species (cucumber, watermelon, *Cucurbita maxima* and *Cucurbita pepo*) were used to construct the phylogenetic tree (**Figure 1A**). Accession numbers of the *ADF* genes in each species were shown in **Supplementary Table 1**. The results showed that 9 CmADFs were distributed in 4 subfamilies and located on six of the 12 melon chromosomes (**Figure 1B**). Two pairs of segmentally duplicated genes *ADF7/10* and *ADF1/4* were detected (**Figure 1B**).

As previously reported (Bowman et al., 2000), there are 5 β-strands and 3 central α-helices in the predicted tertiary structures of CmADFs (**Supplementary Figure 1**). A comparative analysis of the protein sequence and gene structure of the ADFs from melon and *Arabidopsis* showed that they contain the conserved serine-6 residue at the N-terminus (except *CmADF5* and *AtADF5* of which were replaced by threonine, and *CmADF8* of which was deleted), conserved actin binding sites (R98/135/137 and K82/100 in AtADF1) (Dong et al., 2013) (**Figure 1C**), as well as conserved motifs (**Supplementary Figure 2**) and gene structure (**Figure 1D**). Exon-intron structures of *ADFs* in melon and *Arabidopsis* is very conservative (**Figure 1D**). The homologous *ADFs* of the two species were comprised three exons and two introns (except CmADF8) and same number of amino acids in the exons (except CmADF8 and AtADF1). The first exons of CmADFs and AtADFs in Subclass I and II (except CmADF8 and AtADF1) contained only 3 amino acids (start codon ATG), followed by a longer first intron (**Figure 1D**), while the ones of *CmADFs* and *AtADFs* in Subclass III and IV increased to 21 and 24 amino acids due to the alteration of the intron conserved clipping site (GT) after ATG (**Figure 1D**) and intron sliding (Nan et al., 2017).

Analysis of promoter sequences showed that in addition to many light response elements in all members, *CmADF* promoters contained several key defense and stress responsiveness, low-temperature responsiveness, and heat stress cis-acting elements and elements involved in the response to various hormones, such as abscisic acid (ABA), salicylic acid (SA), gibberellins (GA), auxin (IAA), ethylene, and methyl jasmonate (MeJA) (**Supplementary Figure 3**). It also contained DRE and MYB binding sites.

### Expression pattern analysis of *CmADFs* in different oriental melon tissues

**Figure 2:**
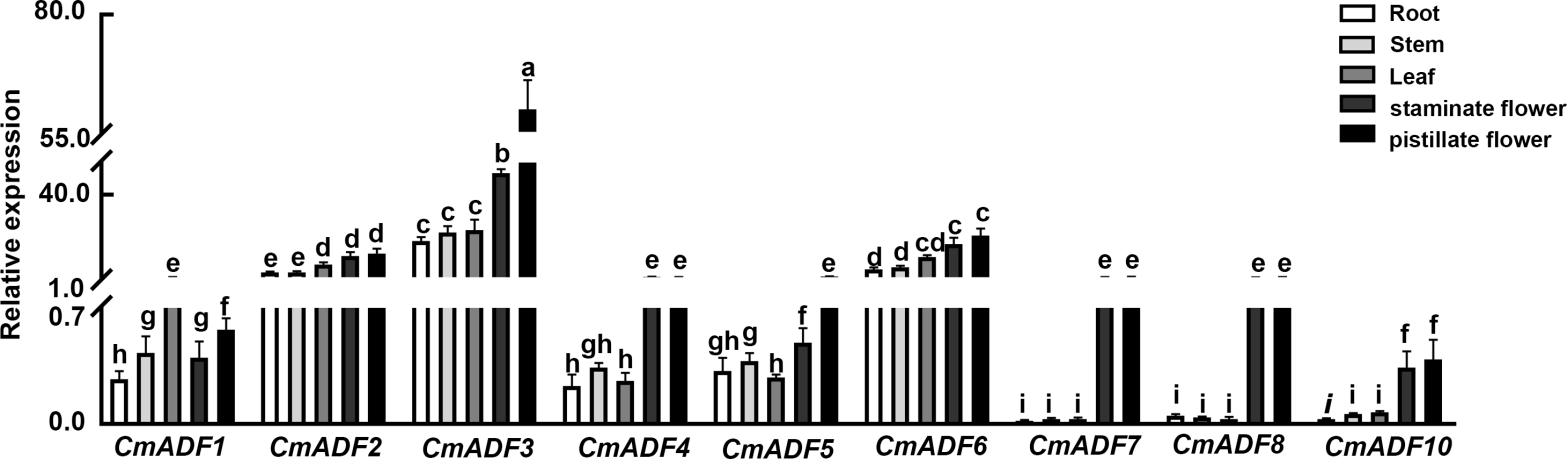
The expression of *CmADFs* in different Oriental melon tissues. The relative expression of *CmADFs* were determined by RT-qPCR, and *18S* was used as an internal control. All values used for statistical analysis were the mean ± SD of three independently replicated experiments, and then a Tukey’s *post-hoc* test was performed using one-way ANOVA. Different lowercase letters indicate a significant difference.

RT-qPCR was performed for analyzing the expression of *CmADFs* in different oriental melon tissues. The expression of *CmADFs* was clearly divided into two categories: *CmADFs* from subclass I, III and IV were expressed in all tissues, while *CmADFs* from subclass II were specifically expressed in flowers (**Figure 2**). Only *CmADF1* was highly expressed in leaves, the other *ADFs* showed higher expression levels in male flowers and female flowers than that in roots, stems, and leaves. Compared with other *CmADFs*, *CmADF2*, *CmADF3* and *CmADF6* had extremely higher expression levels in all tissues.

### Expression pattern analysis of *CmADFs* under low and high temperature stress

**Figure 3:**
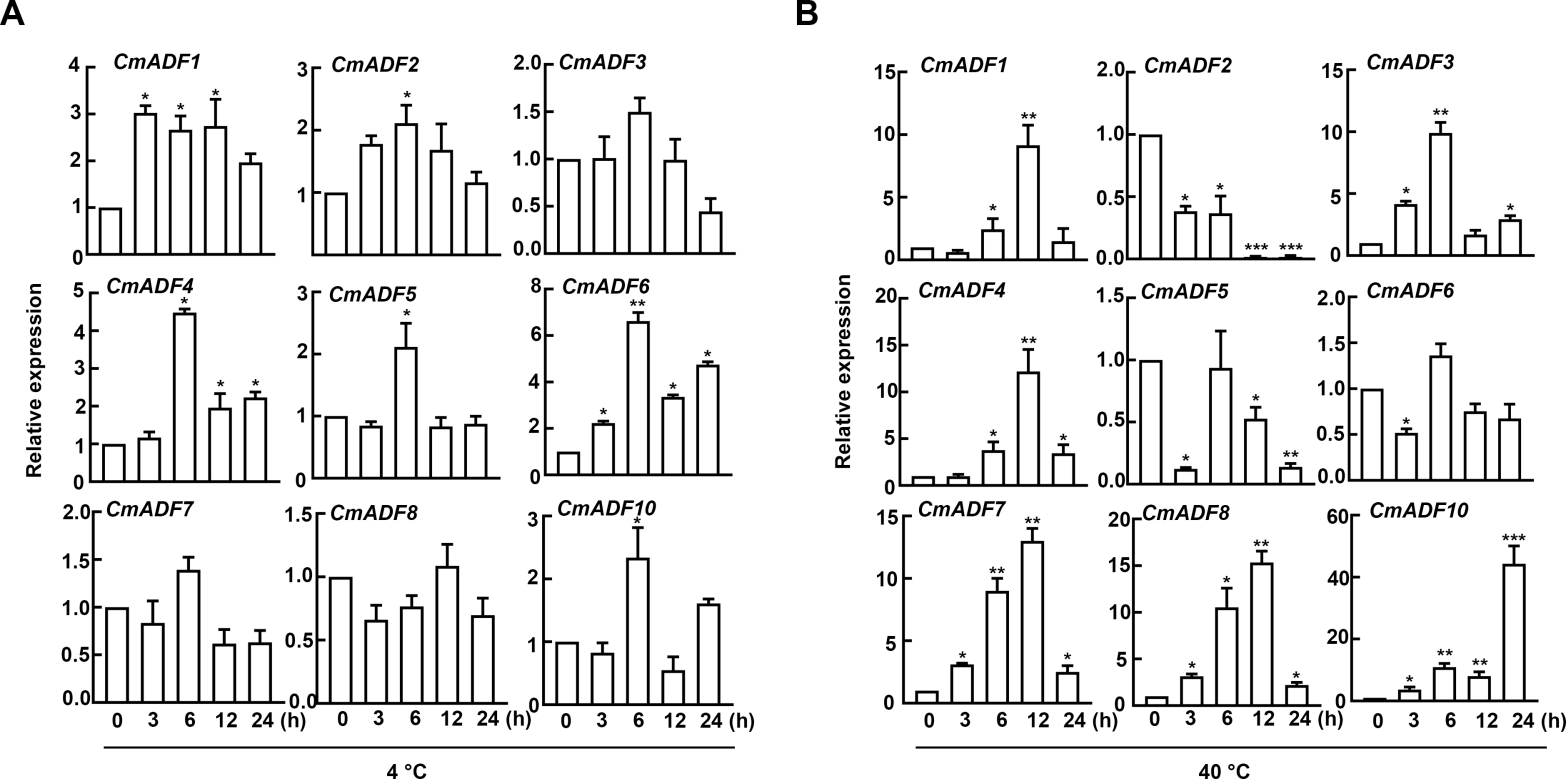
The expression of *CmADFs* under low (4°C) **(A)** and high (40°C) **(B)** temperature treatments according to RT-qPCR. *18S* was used as an internal control. The values are means ± SD from three independent replicate experiments (Student’s t-test, *P < 0.05,** p < 0.01,***P < 0.001). The significant difference is represented by asterisks.

Temperature change is one of the main environmental stressors affecting the growth and yield of melon (Korkmaz and Dufault, 2004). In order to gain an insight into the potential function of CmADFs in unfavorable temperature conditions, we analyzed the expression of *CmADFs* in leaves of oriental melon seedlings at two-leaf stage under low and high temperature stress. The results showed that except for CmADF3/7/8, the expression of other CmADFs was induced by low temperature stress (**Figure 3A**). Among them, *CmADF6* expression was the highest, and *CmADF1* expression was high and stable. Under high temperature stress, the expression of *CmADF1/3/4/7/8/10* was significantly induced, and the expression of *CmADF10* increased the highest, while the expression of *CmADF2/5/6* decreased, and *CmADF2* continued to be significantly down-regulated, indicating that the expression of *CmADFs* was complex under high temperature stress (**Figure 3B**). Under low and high temperature, the expression of *CmADF1/4*/*10* was all induced, but only *CmADF1* was significantly up-regulated.

### Expression pattern analysis of *CmADFs* in ABA and SA stress

**Figure 4:**
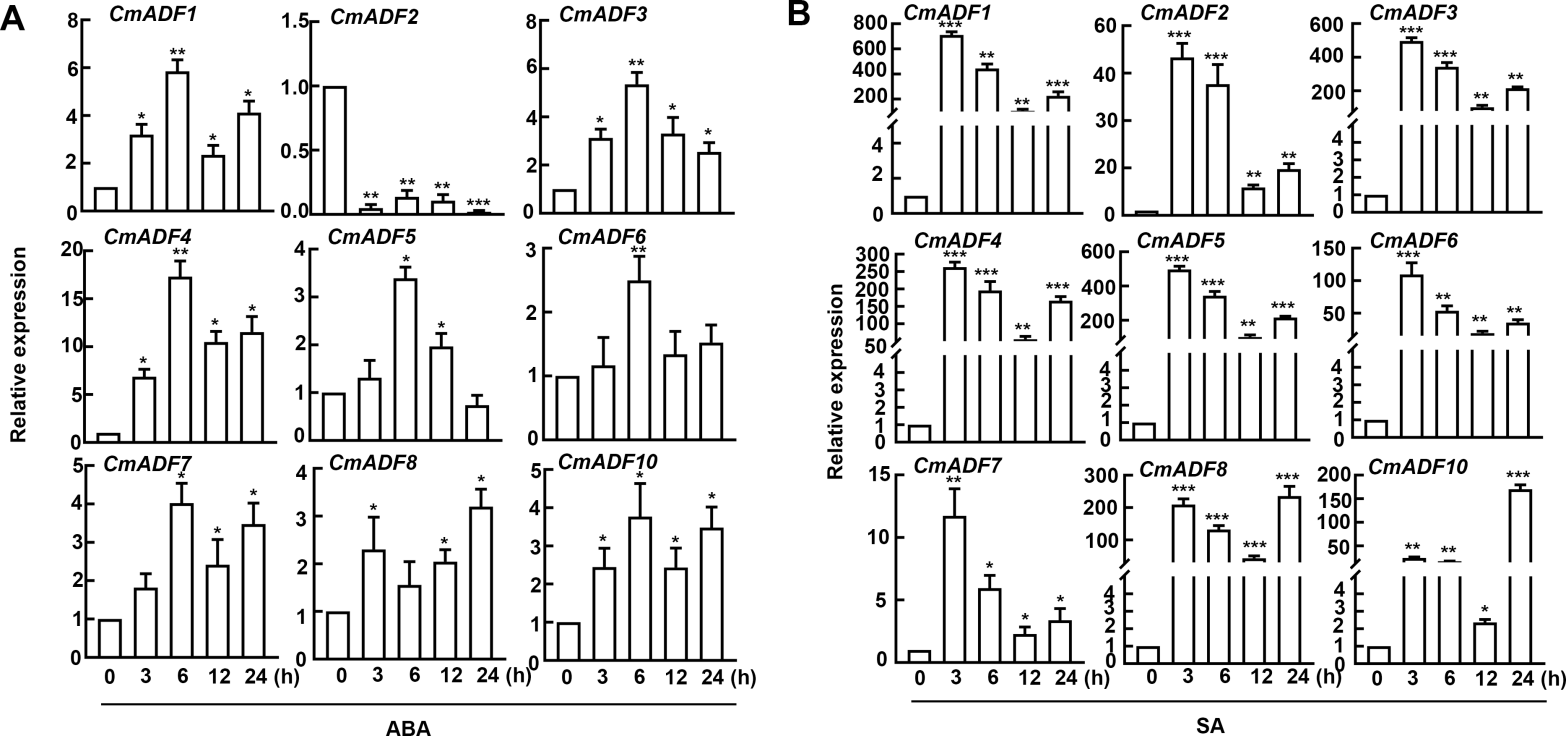
The expression of *CmADFs* under ABA (0.1 mM) **(A)** and SA (0.1 mM) **(B)** treatments according to RT-qPCR. *18S* was used as an internal control. The values are means ± SD from three independent replicate experiments (Student’s t-test, *P < 0.05,** p < 0.01,***P < 0.001). The significant difference is represented by asterisks.

Considering that ABA and SA are the main hormones in plant adaptation to stresses, we analyzed the expression of *CmADFs* in leaves under ABA and SA stress. The results showed that after ABA treatment, only *CmADF2* expression was down-regulated, and all the other *CmADFs* were up-regulated. Other genes reached the highest expression level at 6 h after treatments, except for *CmADF8*, which reached the peak value at 24 h (**Figure 4A**). Under SA stress, all *CmADFs* responded sharply at the beginning, increasing their expression with approximately 13-770 folds, and continued to be highly expressed until 24 h (**Figure 4B**). These results reveal that *CmADFs* respond to SA and ABA induction. Overall, CmADF1 was significantly upregulated in response to temperature and hormonal signals. At the same time, *CmADF1* expression levels was high and stable under low temperature stress, indicating that CmADF1 plays an important role in environmental and hormonal signals. Therefore, CmADF1 was selected for the study of low temperature tolerance in plants.

### Subcellular localization of *CmADF1*

**Figure 5:**
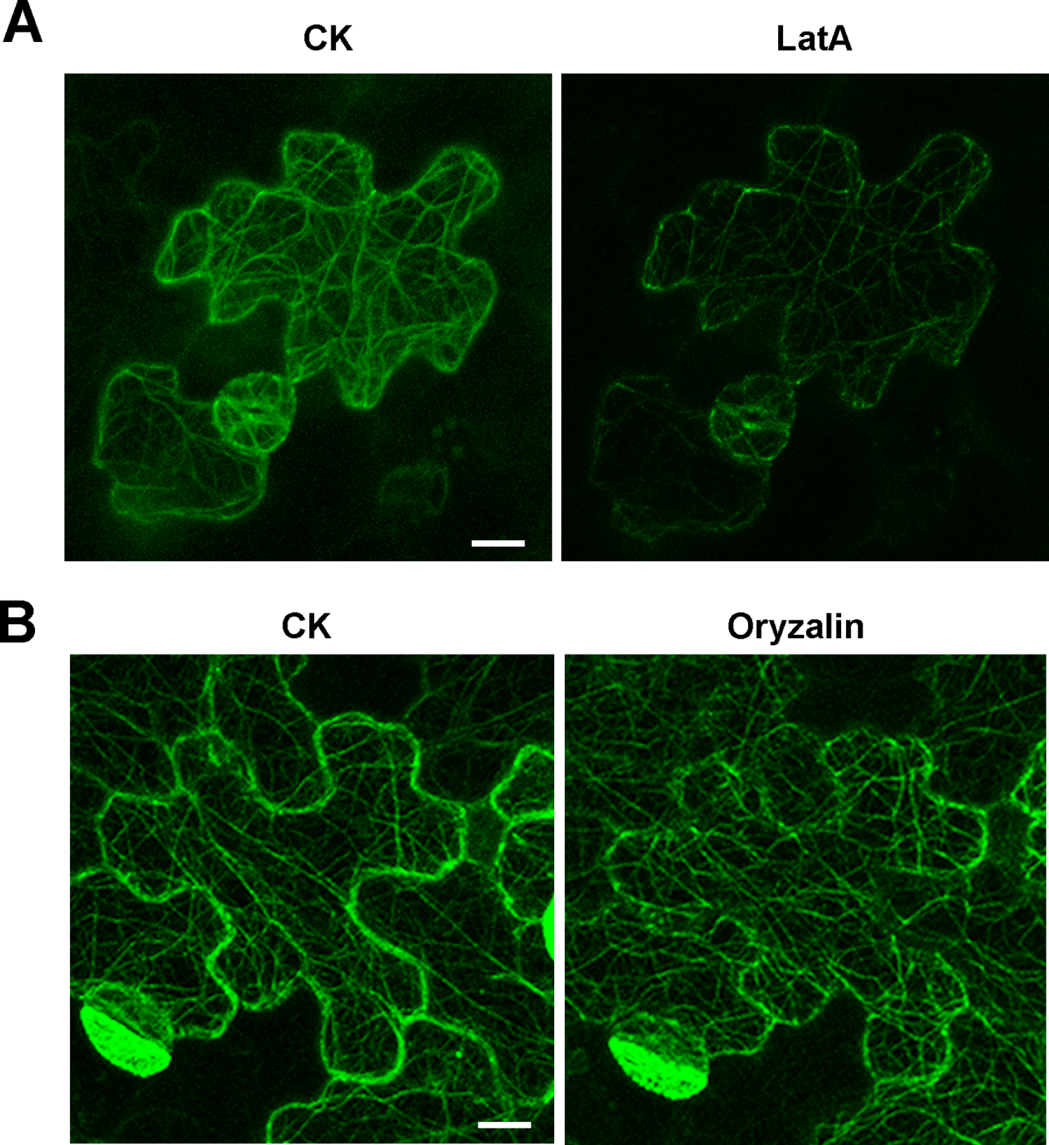
*CmADF1* localization on actin filaments in melon. The leaves of 5-day transgenic *Arabidopsis* homozygous T3 plants were used for observation. **(A)** The actin filaments skeleton formed by *CmADF1*-GFP before (CK) and after Lat B treatment (50 nM). **(B)** The actin filaments skeleton formed by *CmADF1*-GFP before (CK) and after oryzalin treatment (50 nM). Scale bar = 25 μm.

Transgenic *Arabidopsis* plants in T3 were observed by laser scanning confocal microscope for the localization and actin filaments binding of *CmADF1*. As shown in **Figure 5**, a large number of actin filaments bundle structures formed by *CmADF1*-GFP green fluorescent protein in the paver cells in the control group (**Figures 5A, B**). These actin filaments in the paver cells were shortened or disappeared after 50 nM LatB (microfilament depolymerization drug) treatment (**Figure 5A**). Moreover, the filamentous structure did not change after 50 nM Oryzalin (a microtubule depolymerization drug) treatment (**Figure 5B**), further indicating that CmADF1 specifically binds to actin filaments rather than microtubules in *Arabidopsis*.

### Promoter activity analysis of *CmADF1*

**Figure 6:**
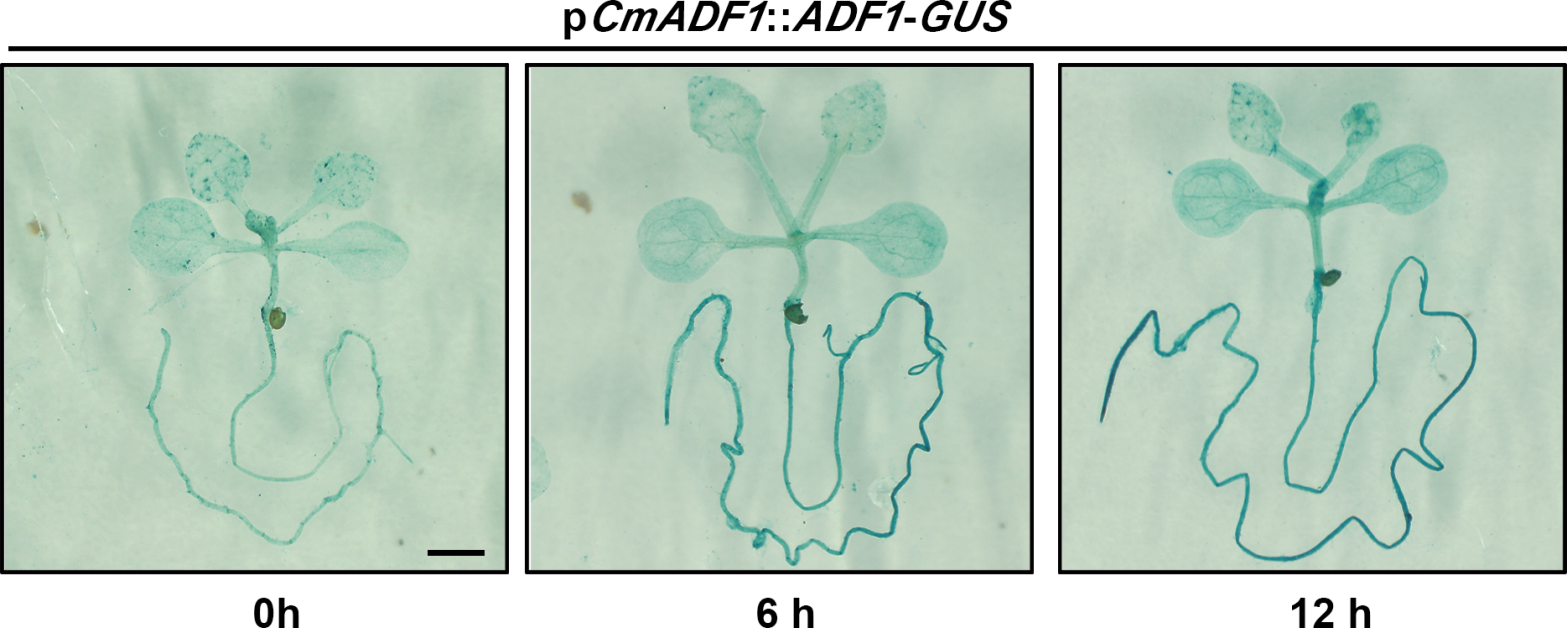
*CmADF1* promoter activity analysis under low temperature treatment. Staining of *pCmADF1*::GUS transgenic *Arabidopsis* plants under low temperature for 0 h, 6 h and 12 h. Scale bar = 1 mm.

It has been reported that *AtADF1* plays an important role in stresses such as salt and high temperature stress (Wang et al., 2021, 2023). Our results showed that *CmADF1* maintained a high expression in melon under low temperature conditions. Therefore, we investigated the mechanism of CmADF1 in melon adaptation to low temperature stress. Firstly, GUS staining was used to detect the effect of low temperature on the activity of CmADF1 promoter, and to further verify the expression pattern of *CmADF1* gene under low temperature stress. After 4°C treatment, the color of p*CmADF1*::GUS plants deepened with the prolongation of treatment time, and the expression of *CmADF1* was significantly up-regulated (**Figure 6**), demonstrating that low temperature promoted the activity of CmADF1 promoter.

### *CmADF1* overexpression affects actin filaments stability under low temperature stress

**Figure 7:**
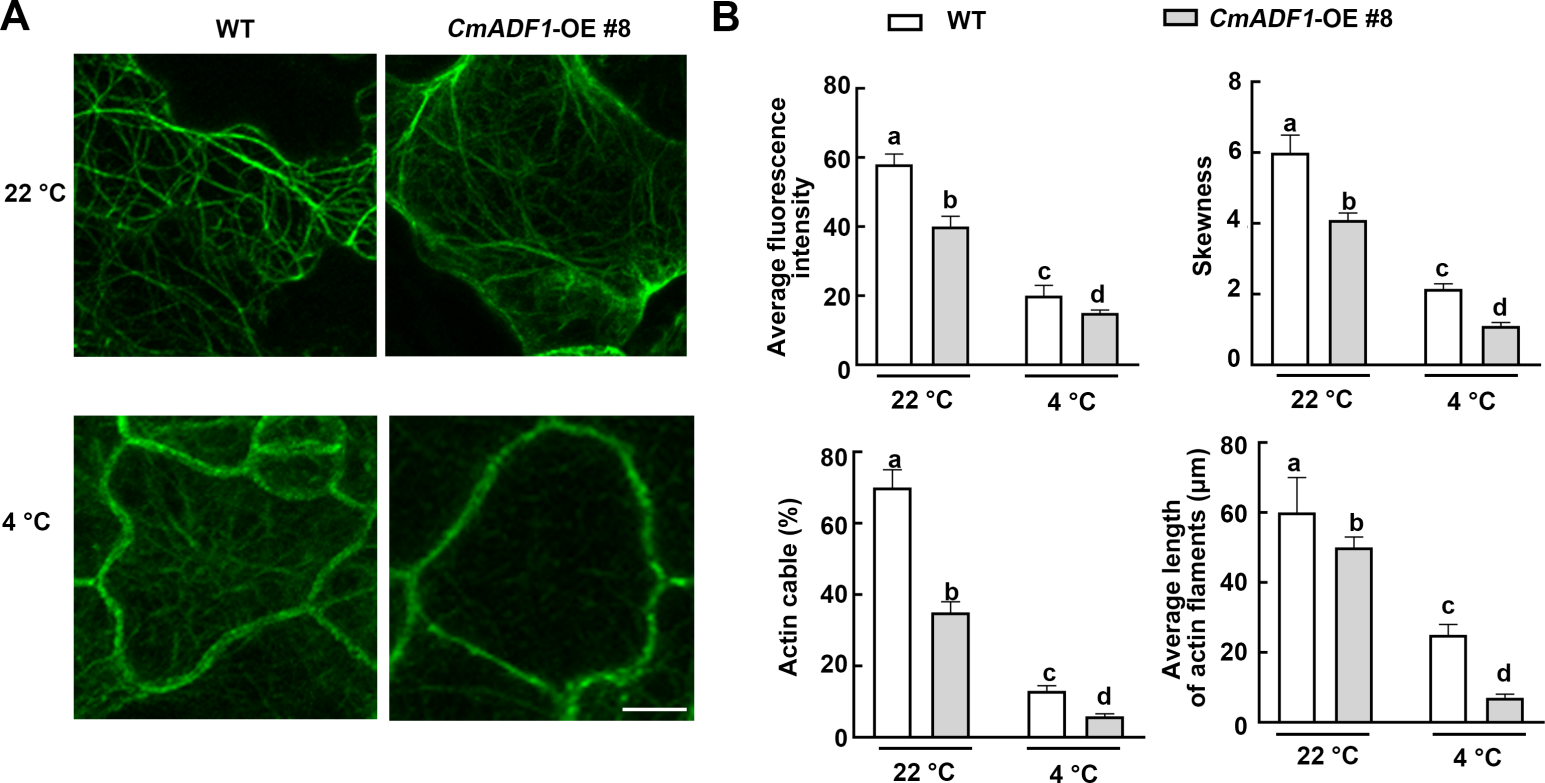
*CmADF1* increases the instability of actin filaments under low temperatures. The organization **(A)**, Average fluorescence density, actin cable and average length **(B)** of actin filaments in WT and *CmADF1*-OE#8 plants under low temperature treatment. Indicators in **(B)** are measured based on images in **(A)**. Values are means ± SD (At least 30 individual seedlings from different genotypes and treatments were used to collect more than 300 images). One-way ANOVA followed by a Tukey’s *post-hoc* test is used for statistical analysis. Different lowercase letters denoted significant differences. Scale bar= 25 μm.

ADF1 is an actin filament depolymerizing protein. To explore whether *CmADF1* can regulate actin filaments under low temperatures, we constructed transgenic *Arabidopsis* overexpressed T3 homozygous lines (*CmADF1*-OE#6 and *CmADF1*-OE#8) (**Supplementary Figure 4**). The homozygous offsprings (*CmADF1*-OE#8) of *CmADF1*-OE#8 × *fABD2-GFP* were selected to observe actin filaments. Compared with WT, *CmADF1*-OE#8 seedlings had fewer actin filaments bundles, and more short filaments under both normal and low temperatures (**Figures 7A, B**). Consistent with the morphology of actin filaments, quantitative analysis of actin filament organization showed that the skewness value, bunting rate, fluorescence density and length of actin filaments in *CmADF1*-OE#8 were significantly lower than those in WT (**Figure 7B**), indicating that overexpression of *CmADF1* caused the instability of actin filaments. Compared with normal conditions, the actin filaments in WT were shorter and finer under low temperature, indicating that low temperature induced the instability of intracellular actin filaments.

### *CmADF1* overexpression enhance the low temperature tolerance in Arabidopsis

**Figure 8:**
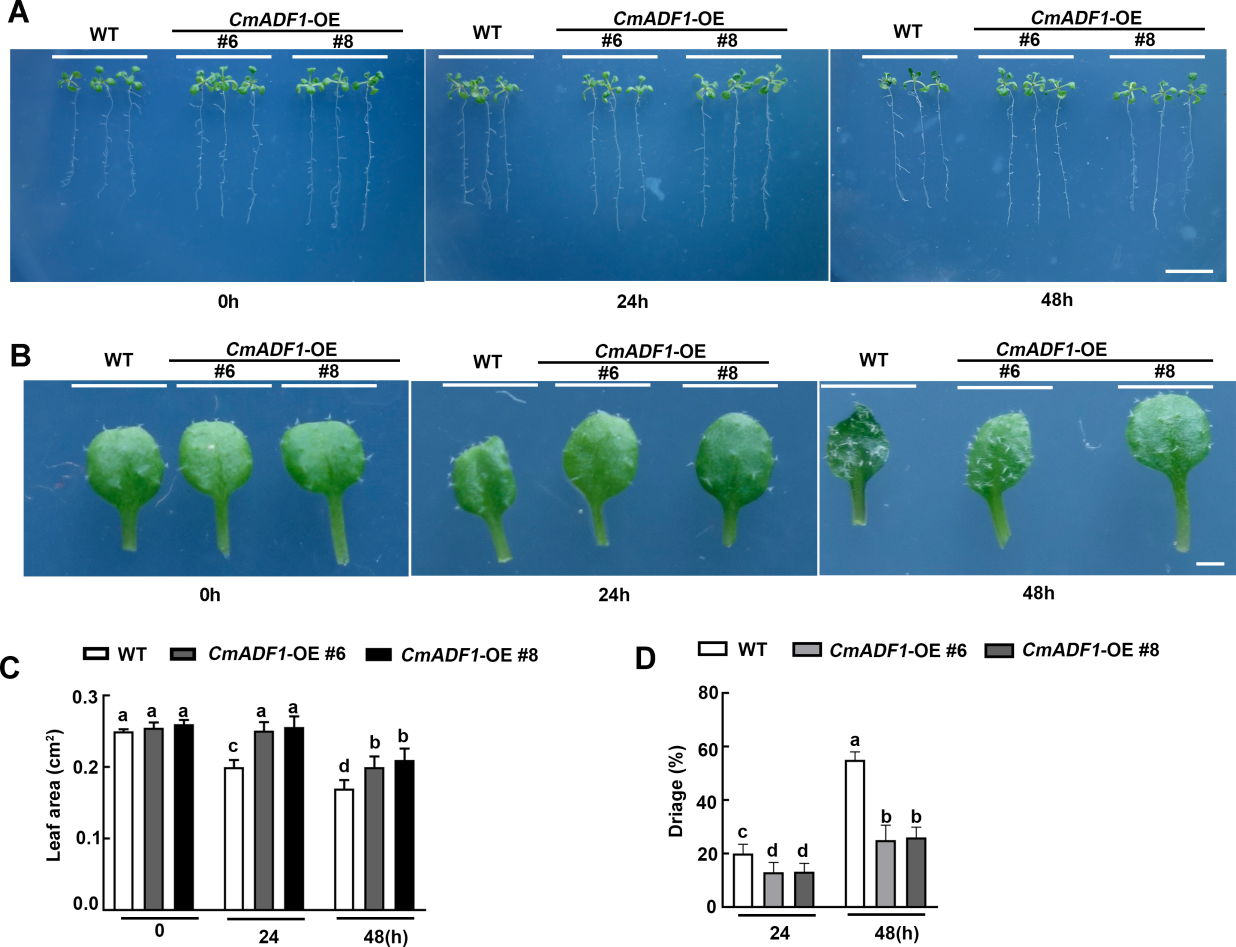
*CmADF1*-OE positively regulates low temperature tolerance of *Arabidopsis*. Plant Phenotype **(A)**, Leaves Phenotype **(B)**, Leaves Area **(C)**, Water loss rate of plants **(D)** of *CmADF1* transgenic seedlings under low temperature treatment. 14-day-old seedlings of WT, *CmADF1*-OE#6 and *CmADF1*-OE#8 were placed in normal temperature (22°C) and low temperature (4°C) chamber for 0, 24 and 48 h. At least 60 leaves from 30 seedlings were measured in **(C)** and at least 400 seedlings were measured in **(D)**. One-way ANOVA followed by a Tukey’s *post-hoc* test is used for statistical analysis. Different lowercase letters denoted significant differences. Scale bar=1cm in **(A)**, Scale bar=1mm in **(B)**.

T3 *CmADF1*-OE#6 and T3 *CmADF1*-OE#8 seedlings were used to analyze the function of CmADF1 under low temperature (**Figure 8**). The phenotypes of 14-day-old WT, *CmADF1*-OE#6 and *CmADF1*-OE#8 seedlings were observed under normal (22°C) and low temperature (4°C) stress for 0, 24 and 48 h. There was no significant difference between WT and overexpressed plants before low temperature treatment. After treatment for 24 and 48 h, the leaves of WT plants shrunk significantly in size (**Figures 8A–C**) and lost more water (**Figure 8D**), compared with those of overexpressed plants. WT plants were more severely damaged than overexpressed plants after 48 h of treatment, with water-soaked spots on the leaves (**Figure 8C**).

*CmADF1*-OE seedlings showed superior resistance by less damage to low temperature stress with more fine actin bundles and short filaments than WT seedlings (**Figures 7A, B**), which implicated that *CmADF1* enhanced plant tolerance to low temperature by regulating actin filaments organization.

*CmADF1*-Silenced Plants are Sensitive in response to Low Temperature

**Figure 9:**
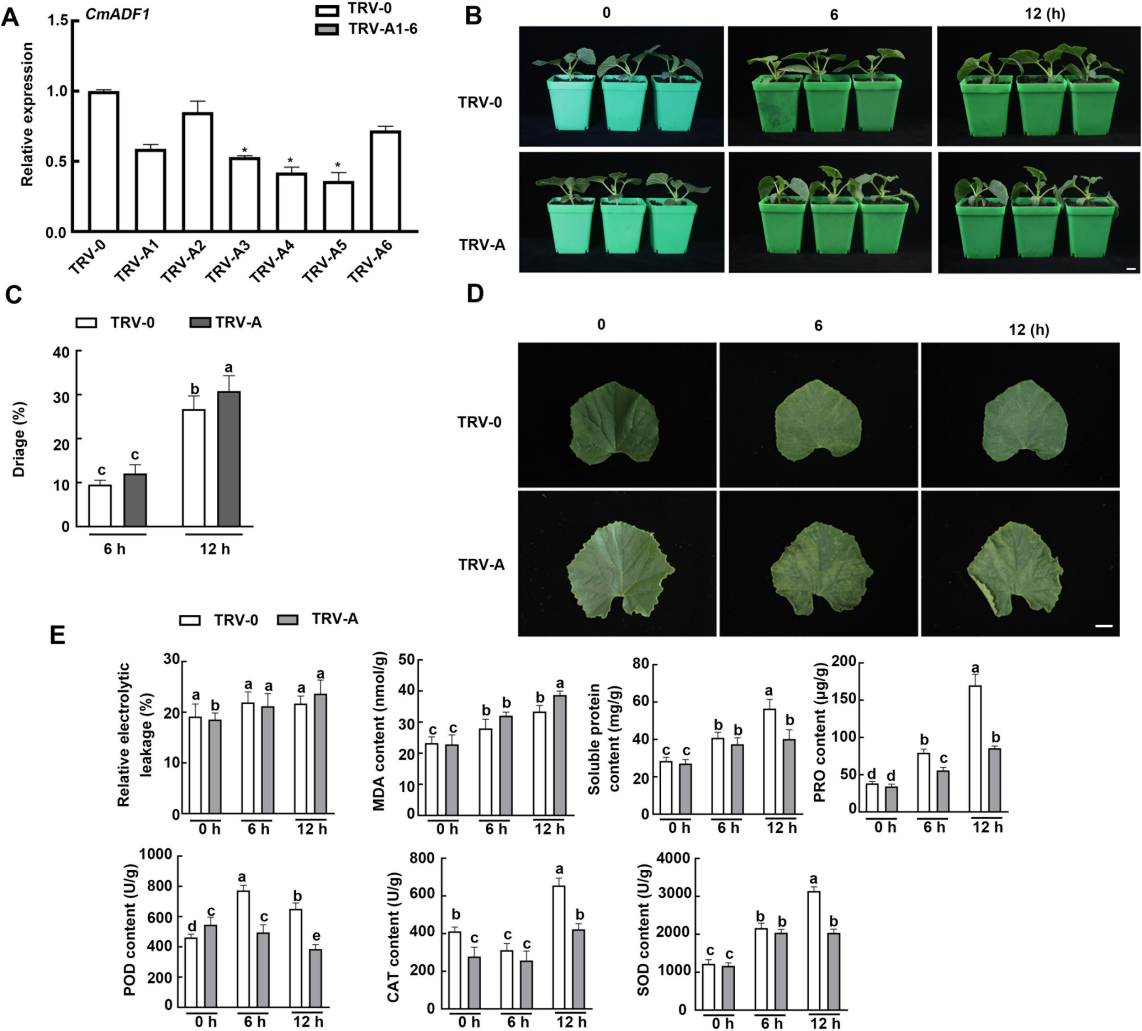
The silencing of *CmADF1* in oriental melon seedlings by virus-induced gene silencing (VIGS) increased its sensitivity to low temperature. *CmADF1* expression **(A)**, Plant phenotype **(B)**, Water loss rate of leaves **(C)**, Leaves **(D)**, the relative electrolyte leakage (REL), malondialdehyde (MDA), Soluble protein and proline content (PRO), SOD, POD and CAT activity **(E)** of TRV*-*0 (control) plants and TRV-A (*CmADF1*-silenced) plants under low temperature treatment. Scale bar=1 cm.

The expression level of *CmADF1* was detected by RT-qPCR when the *CmADF1* gene silencing (TRV-A) plants obtained by VIGS technology had two leaves, and TRV-A plants with high silencing efficiency were selected for subsequent research (**Figure 9A**). Under optimal temperature control conditions, no significant difference in growth between TRV-A and control plants was observed (TRV-0) (**Figure 9B**). After treatment at 4°C for 6 and 12 h, TRV-A plants suffered more serious damage than TRV-0 plants, and their leaves shrunk more seriously and lost more water (**Figures 9B–D**). Compared with TRV-0 plants, the relative electrolyte leakage (REL) and malondialdehyde content of TRV-A plants were higher, while the contents of soluble protein and proline and the activities of SOD, POD and CAT were lower, reaching a significant level at 12 h (except for REL) (**Figure 9E**), illustrating that silencing *CmADF1* reduced the low temperature tolerance of oriental melon.

## Discussion

### Highly conservative *CmADF* genes in oriental melon

Since the *ADF* gene was discovered in the early 1980s, more and more ADFs had been found gradually in different species. In eukaryotic cells, ADFs were encoded by polygene families, which were abundant in plants (Maciver and Hussey, 2002). The reported plant ADF families includes 11 *ADFs* in *Arabidopsis* (Ruzicka et al., 2007), rice (Feng et al., 2006) and tobacco (Khatun et al., 2016), 13 *ADFs* in maize (Huang et al., 2020), 25 *ADFs* in wheat (Xu et al., 2021) and 18 *ADFs* in soybean (Sun et al., 2023). The ADF gene family is considered to be structurally and functionally conserved in plants (McCurdy et al., 2001). In our study, 9 *ADF* genes were identified in oriental melon for the first time, and they were distributed to four subclasses as in *Arabidopsis*. Moreover, they were almost the same as their homologue AtADFs in intron-exon structure, number of amino acids contained in exons, as well as type and number of motifs. There were also some important conserved sites in CmADFs, such as the serine at position 6 of N-terminal (S6), actin binding sites (**Figure 1C**). The activity of ADF protein was regulated by phosphorylation of N-terminal conserved serine or threonine. The S6 mutant of ZmADF3 lost its ability to bind to G-actin and F-actin (Smertenko et al., 1998). The S6 activity of LlADF1 decreased after phosphorylation, and the binding and disassembly F-actin activities were lost (Allwood et al., 2002). The base residues (K82, R135, R137) on β-chain and α-helix form actin binding sites, which is important for G-and F-actin binding (Dong et al., 2013). These results indicate that *ADF* family genes are structurally conserved.

The ADFs in flowering plants probably evolved from a common ancestor (Nan et al., 2017). Fragments and tandem gene duplication are considered to be the major driving forces in the evolution of large gene families (Cannon et al., 2004). Duplicate gene pairs in *Arabidopsis* and wheat are likely caused by tandem duplication, while two pairs of segmentally duplicated genes *ADF7/10* and *ADF1/4* were observed (**Figure 1B**; **Supplementary Table 4**) in melon as in tomato (Khatun et al., 2016), maize (Huang et al., 2020), and soybean (Xu et al., 2021). The ADF gene family has been differentiated in expression pattern and function during a long evolutionary process (Kijima et al., 2016). The angiosperm *ADF* gene family consists of four very conserved subfamilies, which are divided into two classes: reproductive or constitutive/vegetative (Ruzicka et al., 2007). In our study, tissue expression patterns of *ADFs* in melon were similar to those in *Arabidopsis*. *CmADFs* in Subclass I, III, and IV are expressed in all tissues examined and may play a critical role in growth and development (**Figure 2**). *CmADFs* in Subclass II specifically expressed in flowers may contribute to reproductive development (**Figure 2**). Unlike *AtADF8*, which is mainly expressed in roots and root hairs, *CmADF8* is specifically expressed in flowers, indicating that different species are relatively independent in the subsequent evolutionary process. In general, ADF family genes were quite conserved in the long-term evolution of plants.

### A large number of CmADFs respond to temperature stress and hormone signals

Expression analysis of *ADF* gene families in tomato, maize, wheat and soybean revealed that the expression of *ADFs* would change significantly under abiotic stresses such as heat, cold, drought, high salt, abscisic acid (ABA), jasmonic acid (JA) and injury (Khatun et al., 2016; Huang et al., 2020; Xu et al., 2021; Sun et al., 2023). Many ADFs in tomatoes were induced by cold, heat, drought, NaCl, ABA, JA, and injury treatment (Khatun et al., 2016). In maize, ZmADF1 was significantly up-regulated under all abiotic stresses, and ZmADF2 and ZmADF3 were significantly induced under high temperature, drought and ABA treatment (Huang et al., 2020). The expression of *GmADFs* in soybean changed under high temperature, low temperature, drought and salt stresses, and *GmADF2/5/9/12/13/16*/*18* were significantly induced by heat stress (Sun et al., 2023). *TaADF16/17/18* in wheat promoted the freezing resistance of wheat plants acclimated to the cold (Xu et al., 2021). ADF1 in Arabidopsis has been shown to participate in high temperature and salt stresses, and is also the most important member of *Arabidopsis* ADF family involved in stress.

In present study, *CmADF1* was significantly induced in all treatments (**Figures 3**, **4**), suggesting that, similar to *Arabidopsis*, ADF1 in melon may have an important effect on stress tolerance. Under low temperature, *CmADF1* was stably and highly expressed (**Figure 3**), while *AtADF1* was not induced, but *AtADF5* and *AtADF9* in Subclass III were significantly up-regulated (Fan et al., 2016). This indicates that *ADF* genes in different plants are functionally differentiated. Members of a gene family from the same group may have similar functions (Huang et al., 2020). *CmADF1/3/4* from Subclass I and *CmADF7/8/10* from Subclass II were both significantly induced under high temperature, ABA, and SA treatments (**Figures 3**, **4**), suggesting that they may confer plants tolerance to these stresses. Studies have shown that actin depolymerization can increase plant resistance to pathogens, and that SA is crucial to this process (Leontovyčová et al., 2019). The expression of *CmADFs* was increased several hundred-fold under SA treatments, which may enhance resistance to biotic stress by depolymerizing actin filaments dependent on SA signaling pathway. *CmADF2* was sensitive to high temperature and ABA treatments, indicating the diversity and complexity of functions among family members in resistance to stress. A large number of *ADFs* respond to different abiotic stresses, and the function of some of them in stresses has been proven, so it is necessary for us to further study ADFs.

### *CmADF1* plays an important role in plant low temperature tolerance

Low temperature is an important factor affecting the yield of Oriental melon. The detail molecular mechanism of dynamic changes of actin filaments under low temperature is still uncovered, the only thing we know is that low temperature treatment leads to the depolymerization of actin filaments like the other abiotic stresses do, such as salt, high temperature, and osmotic stress (Pokorná et al., 2004; Wang et al., 2011; Fan et al., 2015). Byun et al. (2021) found that DaADF3 functions to depolymerize F-actin into G-actin in transgenic rice plants overexpressing *DaADF3*, and observed cytoskeleton structural changes in *D. antarctica* seedlings in response to cold stress treatment, which imply that DaADF3 regulates the cytoskeleton structure to adapt to changing environmental conditions, especially cold stress in *D. antarctica*. ADF members in Subclass I have functions in resisting biotic/abiotic stresses (Huang et al., 2020). *ADF1* in Subclass I is highly expressed in all tissues and is most closely related to salt stress and high temperature stress (Wang et al., 2021, 2023).

Our study found that *CmADF1* is the only gene in the melon ADF family with highly stable up-regulated under low temperatures (**Figure 3A**), and GUS staining (**Figure 6**) confirmed this result. Therefore, CmADF1 may be a primary protein responding to low temperature stress in the CmADF family. Previous studies have found that some ADFs are involved in responding to low temperature, however there is still a lack of in-depth study on actin filament dynamics. Our studies revealed that low temperature stress induced actin filaments instability (**Figure 8**), which is consistent with the ADF family that is functionally characterized by depolymerization and cutting actin filaments. Our results further indicated that *CmADF1-*OE transgenic seedlings with low temperature-promoted the depolymerization of actin filaments showed more resistant to low temperature (**Figure 7A**). These suggests that the actin filaments morphology of *CmADF1-*OE under low temperature is directly caused by the function of CmADF1 to depolymerize and cut single actin filament, and CmADF1 regulates remodeling of the actin cytoskeleton to adapt to low temperature stress in melon.

Actin filaments depolymerization have been proved to play a positive regulatory role in salt, osmotic, high temperature and drought stress (Wang et al., 2011; Fan et al., 2015). Xu et al. (2021) found that *TaADF16*-OE transgenic *Arabidopsis* plants suffered less freezing damage in comparison with WT, and had higher POD and SOD activities and more soluble sugar accumulation after a 24h incubation at 4°C. They believed that overexpression of *TaADF16* may contributes to the positive effects on ROS scavenging and osmotic regulation, and enhances the freezing resistance of *Arabidopsis* plants. In our experiment, *CmADF1-*OE conferred *Arabidopsis* better growth status under low temperature compared with WT and *Atadf1*, demonstrating that *CmADF1* enhanced the low temperature tolerance of seedlings and promoted seedlings growth. Meanwhile, *CmADF1*-silenced oriental melon seedlings showed that the contents of soluble protein and proline, and the activities of superoxide dismutase (SOD), peroxidase (POD) and Catalase (CAT) were significantly lower than those in the control. Thus, CmADF1 is a key protein that triggers low-temperature induced actin filament depolymerization in melon, which improves the plant’s low-temperature tolerance. Zhang et al. (2021) found that *AtADF5*, as a downstream target gene of C-repeat binding factor (CBF) signaling pathway, is involved in plant response and resistance to low temperature stress by regulating the dynamics of actin filaments. Overexpression of *TaADF16* induces the expression of cold-responsive genes, which may regulate cold tolerance through interaction with ICE (inducer of *CBF* expression)-CBF-related genes (Xu et al., 2021). Synthetic nucleotides designed based on the DRE element contained in the DaADF3 promoter have a high binding affinity with DaCBF7 (Byun et al., 2015, 2021). We also found the DRE binding site in the *CmADF1* promoter (**Supplementary Figure 3**). Whether the CBF protein is an important factor affecting the transcription level of CmADF1 in oriental melon with low temperature tolerance will be our next work. Together, our results demonstrate that CmADF1 plays an important role in plant adaptation to low temperature by leading to depolymerization of actin filaments, providing breakthrough insights into the molecular basis for melon adaptation to low temperature stress.

## Conclusion

In this study, 9 *ADF* genes were identified in Oriental melon, which were clustered into four subfamilies and their proteins contain one conserved ADF-H domain specific to ADF family genes by phylogenetic tree and conserved domain analysis (**Figures 1A, C**). The comparative analysis of *ADFs* in *Arabidopsis* and melon showed that *ADFs* of these two species were highly similar in phylogenetic evolution, tertiary structure, conserved motifs and key conserved sites binding to actin, indicating that plant ADF genes are very conserved in the long-term evolution process (**Figures 1A, C, D**; **Supplementary Figures 1**, **2**). Various *CmADFs* displayed specific tissue expression patterns (**Figure 2**), some were induced by temperature and hormone signals (**Figures 3**, **4**). *CmADF1/2/4/5/6/10 and CmADF1/3/4/7/8/10* were induced under low/high temperature stress, respectively (**Figure 3**). All *CmADFs* responded to SA and ABA signals (**Figure 4**). These results suggested that *CmADFs* may be involved in melon response to stress. *CmADF1* had high and stable expression levels under low temperature stress (**Figure 3A**). *CmADF1* overexpressing plants promoted the instability of actin filaments and enhanced the resistance growth to low temperature treatments (**Figures 7**, **8**), suggesting that CmADF1 plays an important role in low temperature stress in melon. Because the expression of *CmADF1* gene was significant induced by low temperature, and the *CmADF1* gene promoter contained the binding sites of MYB and CBF (**Supplementary Figure 3**), the key transcription factors in plants tolerance low temperature stress, we speculate the role of CmADF1 in low temperature may be regulated by MYB and/or CBF class transcription factors. We will look for upstream transcription factors of the *CmADF1* gene to explore the molecular mechanisms of CmADF1 in low temperature in future research.

## Data Availability

The original contributions presented in the study are included in the article/**Supplementary Material**. Further inquiries can be directed to the corresponding authors.

## References

[B1] Allwood E. G. Anthony R. G. Smertenko A. P. Reichelt S. Drobak B. K. Doonan J. H.. (2002). Regulation of the pollen-specific actin depolymerizing factor LlADF1. Plant Cell 14, 2915–2927. doi: 10.1105/tpc.005363 12417710 PMC152736

[B2] Andrianantoandro E. Pollard T. D. (2006). Mechanism of actin filament turnover by severing and nucleation at different concentrations of ADF/cofilin. Molecular cell 24 (1), 13–23. doi: 10.1016/j.molcel.2006.08.006 17018289

[B3] Bamburg J. R. Bernstein B. W. (2008). ADF/cofilin. Curr. Biol. CB 18, R273–R275. doi: 10.1016/j.cub.2008.02.002 18397729

[B4] Bi S. Li M. Liu C. Liu X. Cheng J. Wang L.. (2022). Actin depolymerizing factor ADF7 inhibits actin bundling protein VILLIN1 to regulate root hair formation in response to osmotic stress in Arabidopsis. PloS Genet. 18, e1010338. doi: 10.1371/journal.pgen.1010338 36095000 PMC9499291

[B5] Bowman G. D. Nodelman I. M. Hong Y. Chua N. H. Lindberg U. Schutt C. E.. (2000). A comparative structural analysis of the ADF/cofilin family. Proteins. 41, 374–384. doi: 10.1002/1097-0134(20001115)41:3<374::aid-prot90>3.0.co;2-f 11025548

[B6] Byun M. Y. Cui L. H. Lee A. Oh H. G. Yoo Y. H. Lee J.. (2021). Abiotic stress-induced *actin-depolymerizing factor 3* from *deschampsia Antarctica* enhanced cold tolerance when constitutively expressed in rice. Front. Plant Sci. 12. doi: 10.3389/fpls.2021.734500 PMC8506025 34650582

[B7] Byun M. Y. Lee J. Cui L. H. Kang Y. Oh T. K. Park H.. (2015). Constitutive expression of DaCBF7, an Antarctic vascular plant Deschampsia Antarctica CBF homolog, resulted in improved cold tolerance in transgenic rice plants. Plant Sci. 236, 61–74. doi: 10.1016/j.plantsci.2015.03.020 26025521

[B8] Cannon S. B. Mitra A. Baumgarten A. Young N. D. May G. (2004). The roles of segmental and tandem gene duplication in the evolution of large gene families in Arabidopsis thaliana. BMC Plant Biol. 4, 10. doi: 10.1186/1471-2229-4-10 15171794 PMC446195

[B9] Clément M. Ketelaar T. Rodiuc N. Banora M. Y. Smertenko A. Engler G.. (2009). Actin-depolymerizing factor2-mediated actin dynamics are essential for root-knot nematode infection of Arabidopsis. Plant Cell 21, 2963–2979. doi: 10.1105/tpc.109.069104 19794115 PMC2768942

[B10] Daher F. B. Geitmann A. (2012). Actin depolymerizing factors ADF7 and ADF10 play distinct roles during pollen development and pollen tube growth. Plant Signal. Behav. 7, 879–881. doi: 10.4161/psb.20436 22751315 PMC3583979

[B11] Ding Y. Shi Y. Yang S. (2019). Advances and challenges in uncovering cold tolerance regulatory mechanisms in plants. New Phytol. 222, 1690–1704. doi: 10.1111/nph.15696 30664232

[B12] Dong C. H. Tang W. P. Liu J. Y. (2013). Arabidopsis AtADF1 is functionally affected by mutations on actin binding sites. J. Integr. Plant Biol. 55, 250–261. doi: 10.1111/jipb.12015 23190411

[B13] Dong C. H. Xia G. X. Hong Y. Ramachandran S. Kost B. Chua N. H. (2001). ADF proteins are involved in the control of flowering and regulate F-actin organization, cell expansion, and organ growth in Arabidopsis. Plant Cell 13, 1333–1346. doi: 10.1105/tpc.13.6.1333 11402164 PMC135580

[B14] Fan T. T. Ni J. J. Dong W. C. An L. Z. Xiang Y. Cao S. Q. (2015). Effect of low temperature on profilins and ADFs transcription and actin cytoskeleton reorganization in Arabidopsis. Biol. Plant 59, 793–796. doi: 10.1007/s10535-015-0546-6

[B15] Fan T. Wang R. Xiang Y. An L. Cao S. (2016). Heat stress induces actin cytoskeletal reorganization and transcript profiles of vegetative profilins and actin depolymerizing factors (ADFs) in *Arabidopsis* . Acta Physiol. Plant 38, 37. doi: 10.1007/s11738-016-2061-6

[B16] Feng Y. Liu Q. Xue Q. (2006). Comparative study of rice and Arabidopsis actin-depolymerizing factors gene families. J. Plant Physiol. 163, 69–79. doi: 10.1016/j.jplph.2005.01.015 16360805

[B17] Fu Y. Duan X. Tang C. Li X. Voegele R. T. Wang X.. (2014). TaADF7, an actin-depolymerizing factor, contributes to wheat resistance against Puccinia striiformis f. sp. tritici. Plant Journal: Cell Molecu Lar Biol. 78, 16–30. doi: 10.1111/tpj.12457 24635700

[B18] Huang Y. C. Huang W. L. Hong C. Y. Lur H. S. Chang M. C. (2012). Comprehensive analysis of differentially expressed rice actin depolymerizing factor gene family and heterologous overexpression of OsADF3 confers Arabido psis Thaliana drought tolerance. Rice (New York N.Y.) 5, 33. doi: 10.1186/1939-8433-5-33 24279948 PMC4883719

[B19] Huang J. Sun W. Ren J. Yang R. Fan J. Li Y.. (2020). Genome-wide identification and characterization of actin-depolymerizing factor (ADF) family genes and expression analysis of responses to various stresses in zea mays L. Int. J. Mol. Sci. 21, 1751. doi: 10.3390/ijms21051751 32143437 PMC7084653

[B20] Huang S. Xiang Y. Ren H. (2011). “Actin-binding proteins and actin dynamics in plant cells,” in The Plant Cytoskeleton, vol. 2 . Ed. Liu B. (Springer-Verlag New York Inc: Springer, New York, NY), 57–80. doi: 10.1007/978-1-4419-0987-9_3

[B21] Hussey P. J. Ketelaar T. Deeks M. J. (2006). Control of the actin cytoskeleton in plant cell growth. Annu. Rev. Plant Biol. 57, 109–125. doi: 10.1146/annurev.arplant.57.032905.105206 16669757

[B22] Inada N. Higaki T. Hasezawa S. (2016). Nuclear function of subclass I actin-depolymerizing factor contributes to susceptibility in arabidopsis to an adapted powdery mildew fungus. Plant Physiol. 170, 1420–1434. doi: 10.1104/pp.15.01265 26747284 PMC4775110

[B23] Khatun K. Robin A. H. Park J. I. Kim C. K. Lim K. B. Kim M. B.. (2016). Genome-Wide Identification, Characteriz- ation and Expression Profiling of ADF Family Genes in Solanum lycopersicum L. Genes 7, 79. doi: 10.3390/genes7100079 27690110 PMC5083918

[B24] Kijima S. T. Hirose K. Kong S. G. Wada M. Uyeda T.Q. (2016). Distinct biochemical properties of arabidopsis thaliana actin isoforms. Plant & cell physiology 57 (1), 47–56. doi: 10.1093/pcp/pcv176 26578694

[B25] Korkmaz A. Dufault R. (2004). Differential cold stress duration and frequency treatment effects on muskmelon seedling and field growth and yield. Eur. J. Hortic. Sci. 69, 12–20.

[B26] Larkin M. A. Blackshields G. Brown N. P. Chenna R. McGettigan P. A. McWilliam H.. (2007). Clustal W and clustal X version 2.0. Bioinf. (Oxford England) 23, 2947–2948. doi: 10.1093/bioinformatics/btm404 17846036

[B27] Leontovyčová H. Kalachova T. Trdá L. Pospíchalová R. Lamparová L. Dobrev P. I.. (2019). Actin depolymerization is able to increase plant resistance against pathogens via activation of salicylic acid signalling pathway. Sci. Rep. 9, 10397. doi: 10.1038/s41598-019-46465-5 31320662 PMC6639534

[B28] Liao J. J. Wang C. H. Xing Q. J. Li. Y. P. Liu X. F. Qi H. Y. (2019). Overexpression and VIGS system for functional gene validation in oriental melon (Cucumis melo var. makuwa Makino). Plant Cell Tissue Organ Culture (PCTOC) 137, 275–284. doi: 10.1007/s11240-019-01568-9

[B29] Liu X. Qin T. Ma Q. Sun J. Liu Z. Yuan M.. (2013). Light-regulated hypocotyl elongation involves proteasome-dependent degradation of the microtubule regulatory protein WDL3 in Arabidopsis. Plant Cell 25, 1740–1755. doi: 10.1105/tpc.113.112789 23653471 PMC3694703

[B30] Lynch M. Conery J. S. (2000). The evolutionary fate and consequences of duplicate genes. Sci. (New York N.Y.) 290, 1151–1155. doi: 10.1126/science.290.5494.1151 11073452

[B31] Maciver S. K. Hussey P. J. (2002). The ADF/cofilin family: actin-remodel ing proteins. Genome Biol. 3, 1–12. doi: 10.1186/gb-2002-3-5-reviews3007 PMC139363 12049672

[B32] McCurdy D. W. Kovar D. R. Staiger C. J. (2001). Actin and actin-binding proteins in higher plants. Protoplasma 215, 89–104. doi: 10.1007/BF01280306 11732068

[B33] Miklis M. Consonni C. Bhat R. A. Lipka V. Schulze-Lefert P. Panstruga R. (2007). Barley MLO modulates actin-dependent and actin-independent antifungal defense pathways at the cell periphery. Plant Physiol. 144, 1132–1143. doi: 10.1104/pp.107.098897 17449647 PMC1914182

[B34] Nan Q. Qian D. Niu Y. He Y. Tong S. Niu Z.. (2017). Plant actin-depolymerizing factors possess opposing biochemical properties arising from key amino acid changes throughout evolution. Plant Cell 29, 395–408. doi: 10.1105/tpc.16.00690 28123105 PMC5354190

[B35] Ouellet F. Carpentier E. Cope M. J. Monroy A. F. Sarhan F. (2001). Regulation of a wheat actin- depolymerizing factor during cold acclimation. Plant Physiol. 125, 360–368. doi: 10.1104/pp.125.1.360 11154343 PMC61016

[B36] Peng S. Q. Huang D. F. (2006). Expression of an Arabidopsis actin-depolymerizing factor 4 gene (AtADF4) in tobacco causes morphological change of plants. J. Plant Physiol. Mol. Biol. 32, 52–56. 16477131

[B37] Pokorná J. Schwarzerová K. Zelenková S. Petrášek J. Janotová I. Čapková V.. (2004). Sites of actin filament initiation and reorganization in cold-treated tobacco cells. Plant Cell Environ. 27, 641–653. doi: 10.1111/j.1365-3040.2004.01186.x

[B38] Qian D. Zhang Z. He J. Zhang P. Ou X. Li T.. (2019). Arabidopsis ADF5 promotes stomatal closure by regulating actin cytoskeleton remodeling in response to ABA and drought stress. J. Exp. Bot. 70, 435–446. doi: 10.1093/jxb/ery385 30476276 PMC6322581

[B39] Roland J. Berro J. Michelot A. Blanchoin L. Martiel J. L. (2008). Stochastic severing of actin filaments by actin depolymerizing factor/cofilin controls the emergence of a steady dynamical regime. Biophys. J. 94, 2082–2094. doi: 10.1529/biophysj.107.121988 18065447 PMC2257902

[B40] Ruzicka D. R. Kandasamy M. K. McKinney E. C. Burgos-Rivera B. Meagher R. B. (2007). The ancient subclasses of Arabidopsis Actin Depolymerizing Factor genes exhibit novel and differential expression. Plant Journal: Cell Mol. Biol. 52, 460–472. doi: 10.1111/j.1365-313X.2007.03257.x 17877706

[B41] Sangwan V. Foulds I. Singh J. Dhindsa R. S. (2001). Cold-activation of Brassica napus BN115 promoter is mediated by structural changes in membranes and cytoskeleton, and requires Ca^2+^ influx. Plant Journal: Cell Mole- Cular Biol. 27, 1–12. doi: 10.1046/j.1365-313x.2001.01052.x 11489178

[B42] Sengupta S. Mangu V. Sanchez L. Bedre R. Joshi R. Rajasekaran K.. (2019). An actin-depolymerizing factor from the halophyte smooth cordgrass, Spartina alterniflora (SaADF2), is superior to its rice homolog (OsADF2) in conferring drought and salt tolerance when constitutively overexpressed in rice. Plant Biotechnol. J. 17, 188–205. doi: 10.1111/pbi.12957 29851294 PMC6330539

[B43] Smertenko A. P. Jiang C. J. Simmons N. J. Weeds A. G. Davies D. R. Hussey P. J. (1998). Ser6 in the maize actin-depolymerizing factor, ZmADF3, is phosphorylated by a calcium-stimulated protein kinase and is essential for the control of functional activity. Plant Journal: Cell Mol. Biol. 14, 187–193. doi: 10.1046/j.1365-313x.1998.00107.x 9669865

[B44] Sun Y. Wang D. Shi M. Gong Y. Yin S. Jiao Y.. (2023). Genome-wide identification of actin-depolymerizing factor gene family and their expression patterns under various abiotic stresses in soybean (*Glycine max*). Front. Plant Sci. 14. doi: 10.3389/fpls.2023.1236175 PMC10413265 37575943

[B45] Tamura K. Stecher G. Peterson D. Filipski A. Kumar S. (2013). MEGA6: molecular evolutionary genetics analysis version 6.0. Mol. Biol. Evol. 30, 2725–2729. doi: 10.1093/molbev/mst197 24132122 PMC3840312

[B46] Tang C. Deng L. Chang D. Chen S. Wang X. Kang Z. (2016). TaADF3, an actin-depolymerizing factor, negatively modulates wheat resistance against puccinia striiformis. Front. Plant Sci. 6. doi: 10.3389/fpls.2015.01214 PMC4716666 26834758

[B47] Tian M. Chaudhry F. Ruzicka D. R. Meagher R. B. Staiger C. J. Day B. (2009). Arabidopsis actin-depolymerizing factor AtADF4 mediates defense signal transduction triggered by the Pseudomonas syringae effector AvrPphB. Plant Physiol. 150, 815–824. doi: 10.1104/pp.109.137604 19346440 PMC2689984

[B48] Wang X. Bi S. Wang L. Li H. Gao B. A. Huang S.. (2020). GLABRA2 regulates actin bundling protein VILLIN1 in root hair growth in response to osmotic stress. Plant Physiol. 184, 176–193. doi: 10.1104/pp.20.00480 32636342 PMC7479883

[B49] Wang L. Cheng J. Bi S. Wang J. Cheng X. Liu S.. (2023). Actin depolymerization factor ADF1 regulated by MYB30 plays an important role in plant thermal adaptation. Int. J. Mol. Sci. 24, 5675. doi: 10.3390/ijms24065675 36982748 PMC10051699

[B50] Wang L. Qiu T. Yue J. Guo N. He Y. Han X.. (2021). Arabidopsis ADF1 is regulated by MYB73 and is involved in response to salt stress affecting actin filament organization. Plant Cell Physiol. 62, 1387–1395. doi: 10.1093/pcp/pcab081 34086948

[B51] Wang C. Zhang L. J. Huang R. D. (2011). Cytoskeleton and plant salt stress tolerance. Plant Signaling Behav. 6, 29–31. doi: 10.4161/psb.6.1.14202 PMC3122001 21301221

[B52] Xing Q. Liao J. Cao S. Li M. Lv T. Qi H. (2020). CmLOX10 positively regulates drought tolerance through jasmonic acid-mediated stomatal closure in oriental melon (*Cucumis melo* var. *makuwa Makino*). Sci. Rep. 10, 17452. doi: 10.1038/s41598-020-74550-7 33060707 PMC7562952

[B53] Xu K. Zhao Y. Zhao S. Liu H. Wang W. Zhang S.. (2021). Genome-wide identification and low temperature responsive pattern of actin depolymerizing factor (ADF) gene family in wheat (*Triticum aestivum* L.). Front. Plant Sci. 12. doi: 10.3389/fpls.2021.618984 PMC7943747 33719289

[B54] Yao H. Li X. Peng L. Hua X. Zhang Q. Li K.. (2022). Binding of 14-3-3κ to ADF4 is involved in the regulation of hypocotyl growth and response to osmotic stress in Arabidopsis. Plant science: an Int. J. Exp. Plant Biol. 320, 111261. doi: 10.1016/j.plantsci.2022.111261 35643603

[B55] Ye J. Zhang W. Guo Y. (2013). Arabidopsis SOS3 plays an important role in salt tolerance by mediating calcium-dependent microfilament reorganization. Plant Cell Rep. 32, 139–148. doi: 10.1007/s00299-012-1348-3 23052592

[B56] Zhang B. Hua Y. Wang J. Huo Y. Shimono M. Day B.. (2017). TaADF4, an actin- depolymerizing factor from wheat, is required for resistance to the stripe rust pathogen Puccinia striiformis f. sp. tritici. Plant Journal: Cell Mol. Biol. 89, 1210–1224. doi: 10.1111/tpj.13459 27995685

[B57] Zhang P. Qian D. Luo C. Niu Y. Li T. Li C.. (2021). Arabidopsis ADF5 acts as a downstream target gene of CBFs in response to low-temperature stress. Front. Cell Dev. Biol. 9. doi: 10.3389/fcell.2021.635533 PMC7876393 33585491

[B58] Zhang Y. P. Yao X. Q. Yang S. J. Xv S. Chen Y. Y. (2017). Effects of low temperature treatment and recovery on the photosynthesis and antioxidantcharacteristics in melon seedlings. Acta Agriculturae Shanghai 33, 41–49.

[B59] Zhang L. Yuan M. Ge Y. Liu Y. Fan J. Ruan Y.. (2010). The microfilament cytoskeleton plays a vital role in salt and osmotic stress tolerance in Arabidopsis. Plant Biol. (Stuttgart Germany) 12, 70–78. doi: 10.1111/j.1438-8677.2009.00201.x 20653889

[B60] Zhao S. Jiang Y. Zhao Y. Huang S. Yuan M. Zhao Y.. (2016). CASEIN KINASE1-LIKE PROTEIN2 regulates actin filament stability and stomatal closure via phosphorylation of actin depolymerizing factor. Plant Cell 28, 1422–1439. doi: 10.1105/tpc.16.00078 27268429 PMC4944410

[B61] Zheng Y. Xie Y. Jiang Y. Qu X. Huang S. (2013). Arabidopsis actindepolymerizing factor7 severs actin filaments and regulates actin cable turnover to promote normal pollen tube growth. Plant Cell 25, 3405–3423. doi: 10.1105/tpc.113.117820 24058157 PMC3809540

[B62] Zhu J. Nan Q. Qin T. Qian D. Mao T. Yuan S.. (2017). Higher-ordered actin structures remodeled by arabidopsis ACTIN-DEPOLYMERIZING FACTOR5 are important for pollen germination and pollen tube growth. Mol. Plant 10, 1065–1081. doi: 10.1016/j.molp.2017.06.001 28606871

