# Supplementary figures and images for: Genome-wide identification of actin-depolymerizing factor family genes in melon (Cucumis melo L.) and CmADF1 plays an important role in low temperature tolerance
Source: Front Plant Sci. 2024 Aug 22;15:1419719. doi: 10.3389/fpls.2024.1419719 (PMC11374638; doi:10.3389/fpls.2024.1419719)

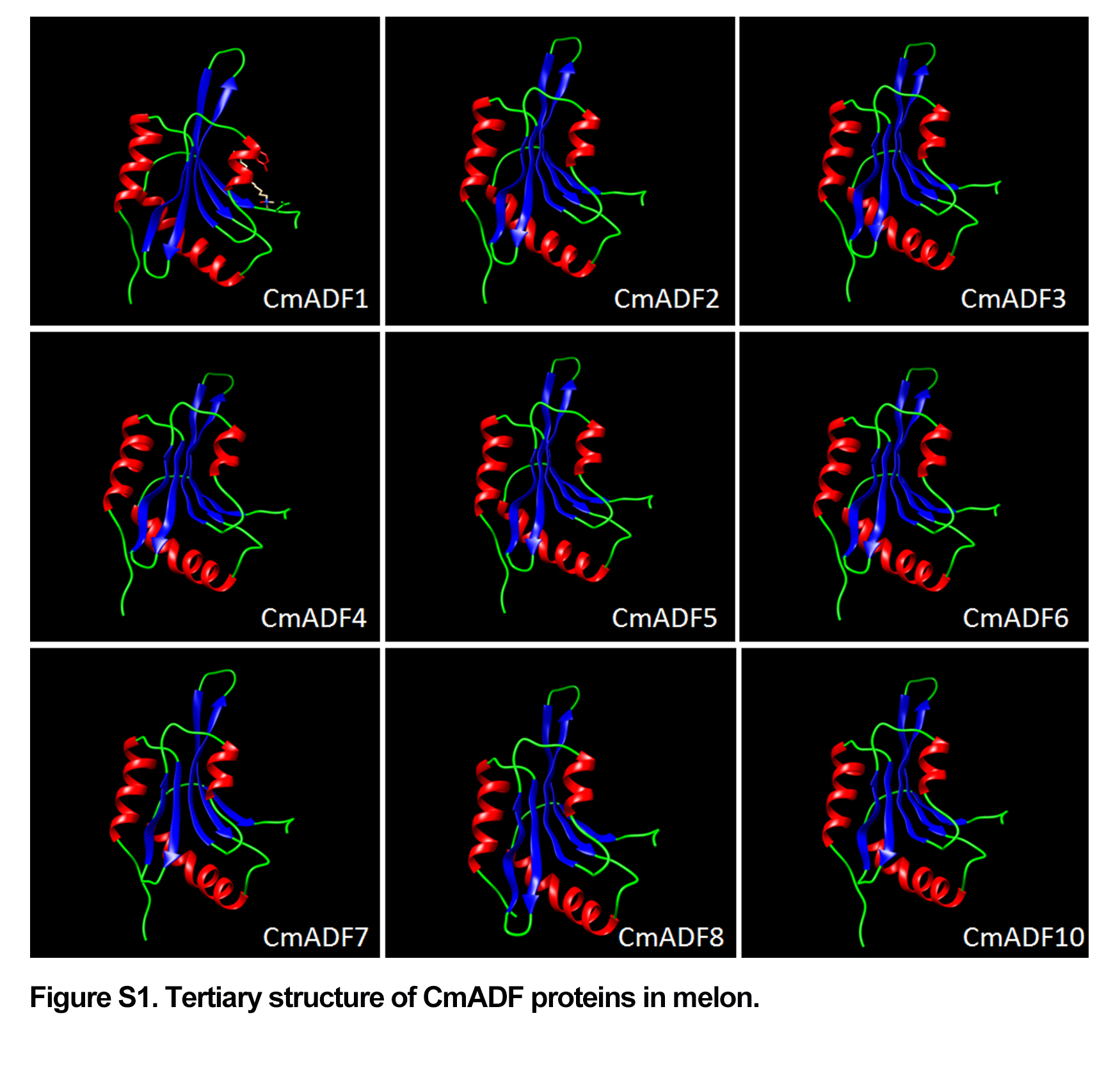

Supplement: Supplementary file 1 [file Image1.tif]

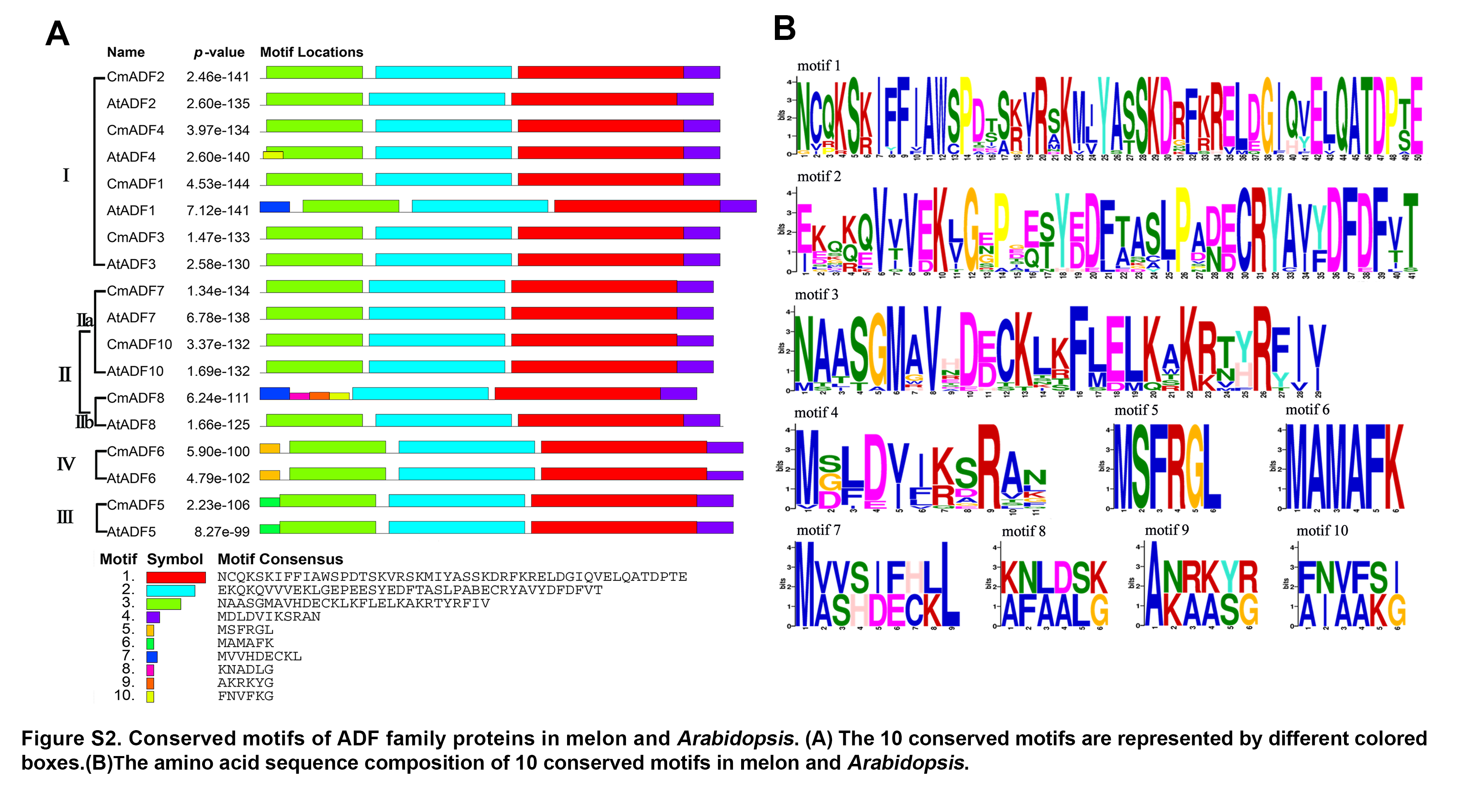

Supplement: Supplementary file 2 [file Image2.tif]

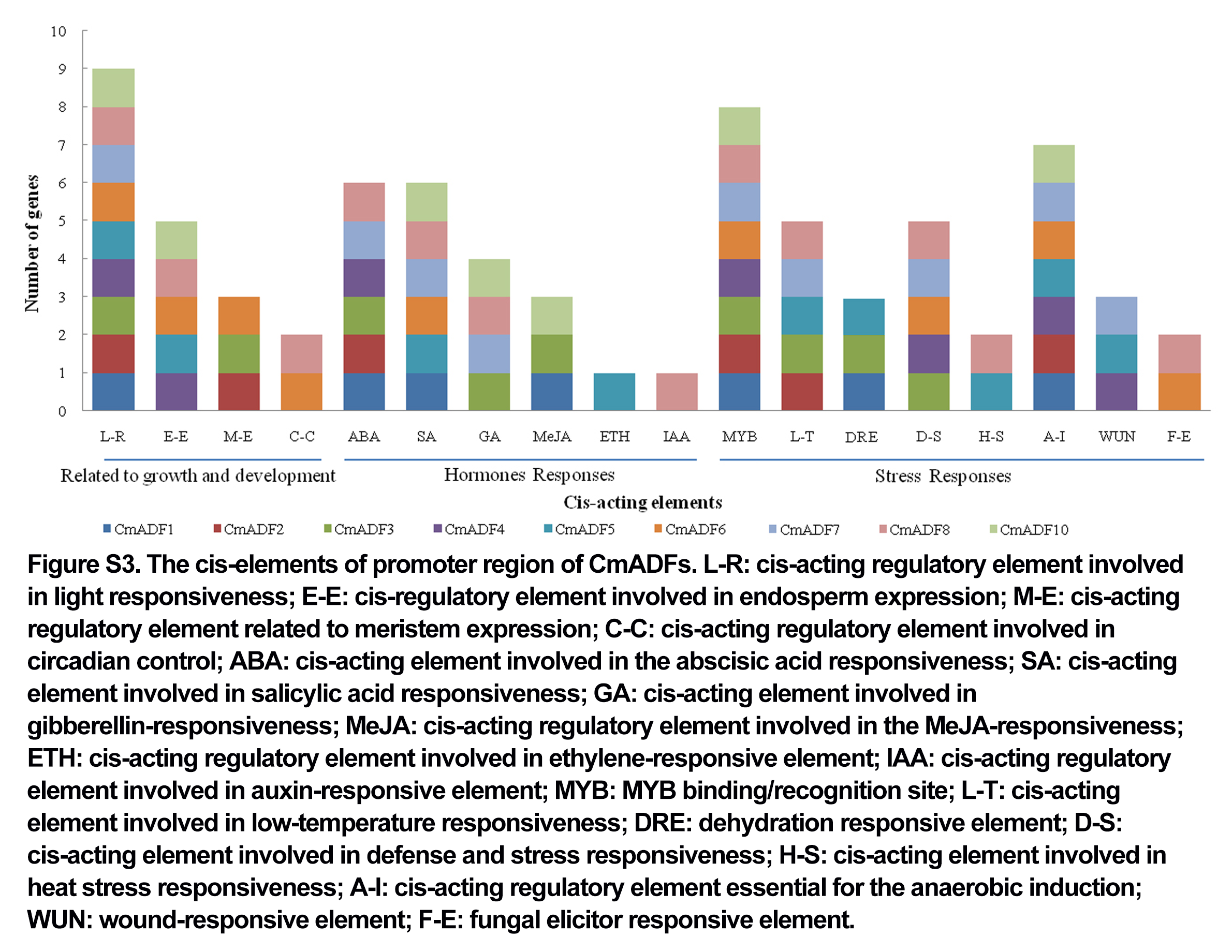

Supplement: Supplementary file 3 [file Image3.tif]

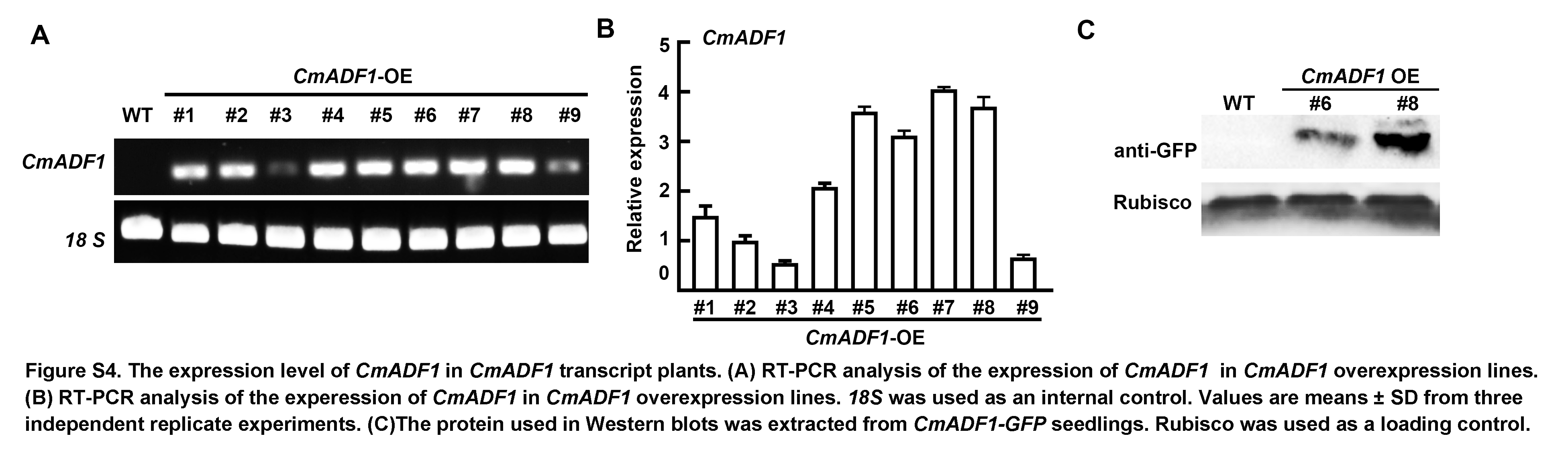

Supplement: Supplementary file 4 [file Image4.tif]
