# Supplementary material for: Genome-wide identification of actin-depolymerizing factor family genes in melon (Cucumis melo L.) and CmADF1 plays an important role in low temperature tolerance
Source: Front Plant Sci. 2024 Aug 22;15:1419719. doi: 10.3389/fpls.2024.1419719 (PMC11374638; doi:10.3389/fpls.2024.1419719)
Supplement: Supplementary file 5 [file Table1.docx]

**Table S1. The information of ADF genes in melon**

| **Gene name** | **Sequence accession No.** | **Chromosome localization**  **and gene direction** | **subclass** | **Amino acid length(aa)** | **Exon number** | **Intron number** | **MW**  **(kDa)** | **pI** | **Subnuclear**  **Localisation** |
| --- | --- | --- | --- | --- | --- | --- | --- | --- | --- |
| CmADF1 | MELO3C003110 | chr8 : 29868870 .. 29871262 (+) | I | 139 | 3 | 2 | 15.93 | 6.60 | Cytoplasm |
| CmADF2 | MELO3C025451 | chr9 : 5516638 .. 5519855 (-) | I | 139 | 3 | 2 | 15.98 | 5.54 | Cytoplasm |
| CmADF3 | MELO3C020711 | chr12 : 2931448 .. 2933570 (-) | I | 139 | 3 | 2 | 16.06 | 6.6 | Cytoplasm |
| CmADF4 | MELO3C009273 | chr4 : 32015472 .. 32017711 (+) | I | 139 | 3 | 2 | 16.09 | 5.64 | Cytoplasm |
| CmADF7 | MELO3C006910 | chr6 : 7200051 .. 7201486 (-) | IIa | 137 | 3 | 2 | 15.82 | 5.32 | Cytoplasm |
| CmADF10 | MELO3C005499 | chr9 : 20858925 .. 20859723 (+) | IIa | 137 | 3 | 2 | 15.94 | 5.33 | Cytoplasm |
| CmADF8 | MELO3C006118 | chr6 : 1169961 .. 1171763 (+) | [IIb](http://melonomics.cragenomica.es/genome/?name=CM3.5_scaffold00006:1169979..1171763) | 132 | 2 | 1 | 15.46 | 5.91 | Cytoplasm |
| CmADF5 | MELO3C020809 | chr11 : 3888401 .. 3889498 (-) | III | 143 | 3 | 2 | 16.41 | 6.73 | Cytoplasm |
| CmADF6 | MELO3C012894 | chr4 : 14115758 .. 14118780 (+) | IV | 146 | 3 | 2 | 17.11 | 7.74 | Cytoplasm |

Chr (represents Chromosome); (−) (represents antisense strand); (+) (represents sense strand); MW (Molecular weight, kD); pI (Isoelectric point).

**Table S2. Accession numbers of ADF gene sequences used in multiple-sequence alignment and phylogenetic analysis**

| **Sequence** | **Accession** | **Sequence** | **Accession** |
| --- | --- | --- | --- |
| CmADF1 | MELO3C003110P1 | AtADF1 | AT3G46010.1 |
| CmADF2 | MELO3C025451P1 | AtADF2 | AT3G46000.1 |
| CmADF3 | MELO3C020711P1 | AtADF3 | AT5G59880.1 |
| CmADF4 | MELO3C009273P1 | AtADF4 | AT5G59890.1 |
| CmADF5 | MELO3C020809P3 | AtADF5 | AT2G16700.1 |
| CmADF6 | MELO3C012894.2P1 | AtADF6 | AT2G31200.1 |
| CmADF7 | MELO3C006910P1 | AtADF7 | AT4G25590.1 |
| CmADF8 | MELO3C006118P1 | AtADF8 | AT4G00680.1 |
| CmADF10 | MELO3C005499P1 | AtADF10 | AT5G52360.1 |
| CsADF1 | Csa4G294380.1 | CmaADF1 | CmaCh03G004390.1 |
| CsADF2 | Csa5G512870.1 | CmaADF2 | CmaCh04G026270.1 |
| CsADF3 | Csa1G480180.1 | CmaADF3 | CmaCh13G001620.1 |
| CsADF4 | Csa3G734880.1 | CmaADF4 | CmaCh06G003100.1 |
| CsADF5 | Csa2G270170.1 | CmaADF5 | CmaCh02G002460.1 |
| CsADF6 | Csa3G389830.1 | CmaADF6 | CmaCh02G017500.1 |
| CsADF7 | Csa3G182120.1 | CmaADF7 | CmaCh02G010680.1 |
| CsADF8 | Csa3G122480.1 | CmaADF8 | CmaCh14G020300.1 |
| ClaADF1 | Cla018229 | CpADF1 | Cp4.1LG19g04390 |
| ClaADF2 | Cla004689 | CpADF2 | Cp4.1LG01g23960 |
| ClaADF4 | Cla017882 | CpADF4 | Cp4.1LG03g02300.1 |
| ClaADF5 | Cla020290 | CpADF5 | Cp4.1LG16g03020.1 |
| ClaADF6 | Cla005983 | CpADF6 | Cp4.1LG05g01070.1 |
| ClaADF7 | Cla006177 | CpADF7 | Cp4.1LG05g06430.1 |
| ClaADF8 | Cla021242 | CpADF8 | Cp4.1LG03g16770.1 |
| ClaADF10 | Cla003643 |  |  |

At, *Arabidopsis thaliana*; Cm, *Cucumis melo*; Cs; *Cucumis sativus*; Cla, *Citrullus lanatus*; Cma, *Cucurbita maxima*; and Cp, *Cucurbita pepo*.

**Table S3. Primers used in this study**

| **Experiments** | **Gene name** | **F(5'-3')** | **R(5'-3')** |
| --- | --- | --- | --- |
| RT-qPCR | CmADF1 | CGAGTGTCGTTATGCTGTCTATG | GGACACCAGGCAATGAAGAA |
|  | CmADF2 | ATAGGTTCAAGAGGGAGTTAGACG | AAATCCACCAAAGCCAAACAACA |
|  | CmADF3 | GAATGCCGATATGCCGTTTATG | GTCAGGAGACCAAGCAATGA |
|  | CmADF4 | TGTAGTGGAGAAAGTCGGTGAG | GACATTCGTCTGAAGGGAGG |
|  | CmADF5 | AATGTGGGTAAGCGACGAGTGTA | GTTTGGTAATGAAGCGGTGAGAT |
|  | CmADF6 | TCCGTGCTAAGATGCTGTATG | GCAGGGTCAGTAGCTTGAAT |
|  | CmADF7 | ACCCATCTTTCTCCTCCTTTCAT | CTCTTCAGGGCTTCCAAGTTTCT |
|  | CmADF8 | TGGGAAGACCAGACGAAACA | CACAGCGTAGCGGCACTCAT |
|  | CmADF10 | CCTGCCAATGAGTGTCGTTAT | GGTGACCAGGCAATGAAGTAA |
|  | Cm18s | AAACGGCTACCACATCCA | CACCAGACTTGCCCTCCA |
| clone | CmADF1 | GGATCCATGGCCAATGCAGCATCAGGA | GTCGACTCAGTTGGCACGGCTTCGAATG |
| Sublcellular localization |  | GGATCCATGGCCAATGCAGCATCAGGAATG | GGTACCTCAGTTGGCACGGCTTCGAATG |
| GUS activity assay |  | AAGCTTGCCTTACGGTGGAGAAGAAAG | GGATCCCTGTCAGATTCACAAAAGAGTGTTC |
| VIGS assay |  | TGAGTAAGGTTACCGAATTCTGATTCAAAGGTCAGAAGCAAGATG | GCGTGAGCTCGGTACCTCAGTTGGCACGGCTTCGAAT |

The enzyme sites are indicated by Underline.

**Table S4. The Duplication events of CmADFgenes in melon genome**

| **Duplicated Gene 1** | **Duplicated Gene 2** | **E-value** | **Identities(%)** | **Positives(%)** | **Ka** | **Ks** | **Ka/Ks** | **Time(Mya)** | **Purifying selection** | **Duplicate type** |
| --- | --- | --- | --- | --- | --- | --- | --- | --- | --- | --- |
| CmADF1 | CmADF4 | 7.00E-88 | 87.05% | 94.24% | 0.7005 | 0.7201 | 0.9729 | 59.025 | Yes | Segment |
| CmADF7 | CmADF10 | 1.90E-89 | 90.51% | 95.62% | 0.4749 | 1.6612 | 0.2859 | 136.164 | Yes | Segment |
